# Eating disorders in minority ethnic populations in Australia, Canada, Aotearoa New Zealand and the UK: a scoping review

**DOI:** 10.1186/s40337-024-01173-y

**Published:** 2025-01-14

**Authors:** A. Williams-Ridgway, R. McGowan, S. McNeil, H. Tuomainen

**Affiliations:** 1 Warwick Medical School, University of Warwick, Coventry, CV47AL UK; 2 Birmingham Women’s and Children’s NHS Foundation Trust, Birmingham, UK; 3 Leicestershire Partnership NHS Trust, Leicester, UK

**Keywords:** Eating disorders, Population groups, Minority ethnic groups, Ethnicity, Treatment, Healthcare disparities

## Abstract

**Background:**

Historically, eating disorder (ED) research has largely focused on White girls and women, with minority ethnic populations underrepresented. Most research exploring EDs in minority ethnic populations has been conducted in the United States (US). The aim of this scoping review, the first of its kind, was to systematically examine research on disordered eating and EDs among minority ethnic populations in Australia, Canada, Aotearoa New Zealand and the United Kingdom (UK), four countries with shared sociocultural and healthcare characteristics. An inequity lens was applied to highlight gaps in research, access, and treatment experiences.

**Method:**

Five databases (Medline, Embase, PsycINFO, CINHAL and Web of Science) were searched up to March 7, 2024. Two independent reviewers screened titles and abstracts and full texts against eligibility criteria resulting in the inclusion of 87 records (76 peer-reviewed articles and 11 theses). Included studies were charted according to their focus, study design, sample characteristics and findings, with a particular focus placed on prevalence, access to treatment and treatment experience.

**Results:**

The majority of identified studies were conducted in the UK (61%, 53 studies). There was a notable lack of studies investigating assessment, diagnosis and intervention. Methodologies varied, though most studies utilised cross-sectional survey designs. Most samples were non-clinical, exclusively or predominantly girls and women, and focused on adolescents and young adults. Asian populations were the most frequently studied minority ethnic group. Understanding of prevalence and treatment experience amongst minority ethnic groups was limited.

**Conclusion:**

There is a need for further research addressing inequities in ED prevalence, service access, and treatment experiences among minority ethnic and Indigenous groups, especially in Australia, Canada and Aotearoa New Zealand. Improved ethnicity data collection and culturally sensitive approaches to assessment, diagnosis and treatment are essential. Recommendations for future research and clinical practice are provided.

**Supplementary Information:**

The online version contains supplementary material available at 10.1186/s40337-024-01173-y.

## Abstract (plain language summary)

More research exploring disordered eating and eating disorders (EDs) in minority ethnic groups is needed outside of the United States (US), where most research has been conducted. We conducted a scoping review to identify studies examining disordered eating and EDs in minority ethnic individuals in Australia, Canada, Aotearoa New Zealand and the United Kingdom (UK). A particular focus was placed on studies which explored prevalence, access to treatment and treatment experiences. We searched five academic databases and screened resulted against inclusion criteria, identifying 87 records (76 peer-reviewed articles and 11 theses). Data on the study focus, design, methods, sample characteristics and findings were extracted. Most studies (61%) were conducted in the UK. The topics covered by studies varied and a wide range of methods were used. Samples were typically non-clinical populations and mostly young girls and women. Asian populations were the minority ethnic group most frequently included in studies. Understanding of prevalence and treatment experience amongst minority ethnic groups was limited. There is a need for further research with underrepresented groups, especially to better understand how these groups access ED services and experience the treatment delivered by services. Efforts to improve the collection and reporting of ethnicity data are also required.

**Supplementary Information:**

The online version contains supplementary material available at 10.1186/s40337-024-01173-y.

## Introduction

Eating disorders (EDs), including anorexia nervosa (AN), bulimia nervosa (BN), binge eating disorder (BED) and other specified feeding or eating disorder (OSFED), are severe and often life-threatening psychiatric disorders [1]. Worldwide lifetime prevalence of EDs is estimated to be 0.91% [2], with especially high prevalence in developed, Western countries [2] and onset typically occurring during adolescence [3], before the age of 25 [4].

The “skinny, White, affluent girl” (SWAG) stereotype [5] and “golden girl phenomenon” [6] have historically framed EDs as primarily affecting young, thin, affluent, cisgender White girls and women. However, current evidence indicates EDs affect people of all ages, body shapes and sizes, socioeconomic statuses (SES), sex, sexual orientations, gender identities and ethnicities [7, 8]. Despite this, the diversity of people affected by EDs is often not reflected in research, which tends to focus disproportionately on homogenous samples of young White girls and women [9].

Prevalence of disordered eating and EDs appears to be comparable across ethnic groups [10–12] and in some cases higher for minority ethnic individuals relative to White individuals [13]. However, the exclusion of minority ethnic individuals from much of the research means relatively little is known about the unique experiences and needs of these populations in relation to EDs. To date, the majority of the limited research exploring disordered eating and EDs in minority ethnic populations has been conducted in the United States (US) [14]. This is evident in several narrative and systematic reviews that focus largely on US-based studies, leaving out global perspectives. The reviews have predominantly explored the prevalence, presentation, risk factors and treatment of EDs among minority ethnic individuals, with the vast majority of included studies originating in the US [15–20].

The applicability of US-based research findings to other countries with different sociocultural contexts, healthcare systems and demographic patterns, however, remains unclear [10, 21]. Further, US-focused research alone does not offer a comprehensive understanding of the diversity of experiences within and across ethnic groups in different national contexts [22]. Consequently, more research exploring disordered eating and EDs in minority ethnic populations conducted outside of the US is needed [14]. This gap in the literature is particularly critical when considering how ethnic and racial inequities in healthcare impact access to and experiences of treatment for EDs.

This scoping review aims to address this gap by identifying and describing studies on disordered eating or EDs among minority ethnic individuals across four countries: Australia, Canada, Aotearoa New Zealand and the United Kingdom (UK). These countries were chosen for their shared cultural, societal, linguistic, and healthcare system similarities, shaped by colonial histories and significant immigration. These commonalities influence their social structures, political systems and ethnic diversity, including the experiences of marginalized groups such as ethnic minorities and Indigenous peoples. Each country has large minority ethnic populations due to high levels of immigration and/or Indigenous displacement [23–26], making them pertinent for examining ethnic disparities in healthcare access and outcomes [27–29]. These countries also all have publicly funded healthcare systems aiming to provide universal access to healthcare, including mental health services [30, 31]. This offers a shared framework for exploring disparities in access to care, particularly in relation to EDs. Additionally, these countries are experiencing rising rates of EDs and have active research and policy initiatives addressing ethnic disparities [32–36], making them ideal for investigating how ethnicity, culture, and healthcare systems intersect to shape access to and outcomes of ED treatment. This review provides a comprehensive overview of the extent, range and nature of research on disordered eating and EDs among minority ethnic groups in these countries. By identifying significant gaps in the literature, it aims to guide future research outside of the US on ethnic and racial differences in ED prevalence, treatment access and outcomes.

Given the documented racial and ethnic inequities in access to and experiences of specialist mental health services [37, 38], including ED services [18], a particular focus will be placed on studies reporting findings regarding prevalence, access to treatment, and treatment experiences. The aim is to identify any disparities in treatment access and outcomes across ethnic groups and to explore potential ways to address these inequities in clinical practice. This focus on equity is especially important given EDs are associated with significant physical and psychiatric morbidity and high mortality rates [39]. Timely and effective diagnosis and intervention are essential for improving treatment outcomes and recovery prospects [40]. Addressing these inequities in care is crucial to reducing the harm and burden caused by EDs, particularly for underserved minority ethnic populations.

### Objectives

The specific objectives of this review are to:

1. Map the literature according to the main themes/topics addressed, methodologies used, and populations targeted.
2. Identify and report on findings from studies examining ED prevalence, access to treatment and treatment experiences and explore ethnic differences in these areas.
3. Identify gaps and/or limitations in the literature and suggest areas for future research.

## Method

### Review methodology

Whereas a systematic review aims to answer well-defined research questions by systematically synthesising and evaluating homogenous studies, the purpose of a scoping review is to summarise an existing body of literature and identify any knowledge gaps [41, 42]. Given the broad aims of the current review and variability of included studies in terms of research design and methods, a scoping review methodology was identified as being most appropriate. This review followed methodology outlined by Arksey and O’Malley [41] and was conducted in accordance with the preferred reporting items for systematic reviews and meta-analyses, extension for scoping reviews (PRISMA-ScR) [43]. As per PRISMA-ScR guidelines, a protocol was developed. The final protocol was registered on Open Science Framework (https://osf.io/w3d6g) and published elsewhere as a peer-reviewed article [44], as such a summary of methods will be provided here.

### Eligibility criteria

Eligibility criteria were developed in an iterative process guided by the Population, Context, Concept framework [45]. Our initial plan was to conduct a global review. However, a preliminary review of database search results and further reading of related review articles highlighted most relevant research was conducted in the US. Time and resource constraints meant including these studies would not have been feasible. Consequently, we refined the scope of the review and aligned the focus on access to treatment and treatment experiences to studies from countries outside of the US with established minority ethnic populations, shared cultural and societal characteristics, and broadly comparable healthcare systems: Australia, Canada, Aotearoa New Zealand and the UK. Additionally, we decided to exclude studies which focused exclusively on Avoidant Restrictive Food Intake Disorder, Pica and Rumination Disorder. These diagnoses were moved to the ED section of the Diagnostic and Statistical Manual of Mental Disorders, Fifth Edition (DSM-5) from the Feeding and Eating Disorders of Infancy and Early Childhood section of the DSM, Fourth Edition, Text Revision (DSM-IV-TR) [46] and have been less commonly explored in relation to racial and ethnic diversity in EDs [16].

Final eligibility criteria are outlined in Table 1. For the purpose of this review, ethnic groups which are numerically smaller and non-dominant in relation to the majority population were considered minority ethnic groups [47]. Studies involving clinical ED patients were included even if they did not specify diagnostic criteria, assuming that individuals would have been assessed by a clinician and had symptoms severe enough to warrant treatment. Studies recruiting participants from multiple countries were considered for inclusion if data were reported separately for each country. Studies involving stakeholders such as clinicians and relatives were also included due to their contribution to knowledge of treatment of EDs in minority ethnic populations. Both peer reviewed published articles and theses were eligible for inclusion. To avoid overlap, where relevant theses were published, the article was included and the theses removed as a duplicate. Due to resource constraints, only articles published in English were included.

**Table 1:** Eligibility criteria

	Inclusion	Exclusion
Population	• Sub- or full sample of minority ethnic participants or data related to minority ethnic individuals. • Where multiple ethnic groups are included in a sample the findings are reported according to ethnicity. For example, ethnicity is the main predictor variable in analyses or the paper reports on ethnic differences in study outcomes (e.g., prevalence rates, scores on psychometric measures, referral rates etc.). • All ages	• Where multiple ethnic groups are included in the sample, but authors did not report any outcomes or analysis according to ethnicity (e.g., ethnicity only reported in sample demographics, analyses reported for sample as a whole rather than individual ethnic groups, where analyses are controlled or adjusted for ethnicity but no further findings or data relating to ethnicity were reported).
Concept	• Study focuses on clinical EDs (i.e., meeting DSM or ICD criteria as assessed using clinician interview or a validated psychometric measure) or disordered eating (i.e., assessed with a validated psychometric measure).	• Focuses solely on risk factors, associated concepts or related conditions (e.g., food addiction, obesity, body image). • Focuses solely on ARFID, Pica or Rumination Disorder.
Context	• Study conducted in Australia, Canada, Aotearoa New Zealand or the United Kingdom.	
Study Design	• Any study design (e.g., case series, case studies, cohort studies, randomized controlled trials etc.)	
Publication Type	• Peer reviewed journal articles or theses.	• Review articles, editorials, commentaries, letters, newsletters, opinion or reflection pieces, book chapters, book reviews, poster presentations or conference abstracts.
Language	• Published in English.	

Abbreviations: ARFID – Avoidant Restrictive Food intake Disorder; DSM – Diagnostic and Statistical Manual of Mental Disorders; ED – eating disorder; ICD – International Classification of Diseases

*From*: Page MJ, McKenzie JE, Bossuyt PM, Boutron I, Hoffmann TC, Mulrow CD, et al. The PRISMA 2020 statement: an updated guideline for reporting systematic reviews. BMJ 2021;372:n71. doi: 10.1136/bmj.n71 [192]. For more information, visit: http://www.prisma-statement.org/

### Search strategy

To identify relevant articles five electronic databases, Medline (Ovid), PsycINFO (Ovid), CINHAL (EBSCO) and Web of Science (Clarivate), were searched on March 30th, 2023. For the sake of completeness a web search was also conducted using Google Scholar. An updated search was conducted on March 7th, 2024. The search strategy was developed by members of the research team (HT, RM and SM) in consultation with a specialist academic support librarian. Search terms were tailored for each database and included a combination of key words and indexed search terms relating to EDs and ethnicity. To ensure the search was as comprehensive as possible broad keywords and search terms were used [41]. No date limits were applied to the search results. The full search strategy for each database is available in Additional File 1.

Though the focus of the review was revised, the original search strategy was not altered. Given the search strategy was designed to be comprehensive and identify articles from all countries and on all types of disordered eating behaviours and EDs, it was felt that all articles relevant to this review should have been captured by the initial search strategy.

### Selection process

**Fig. 1:**
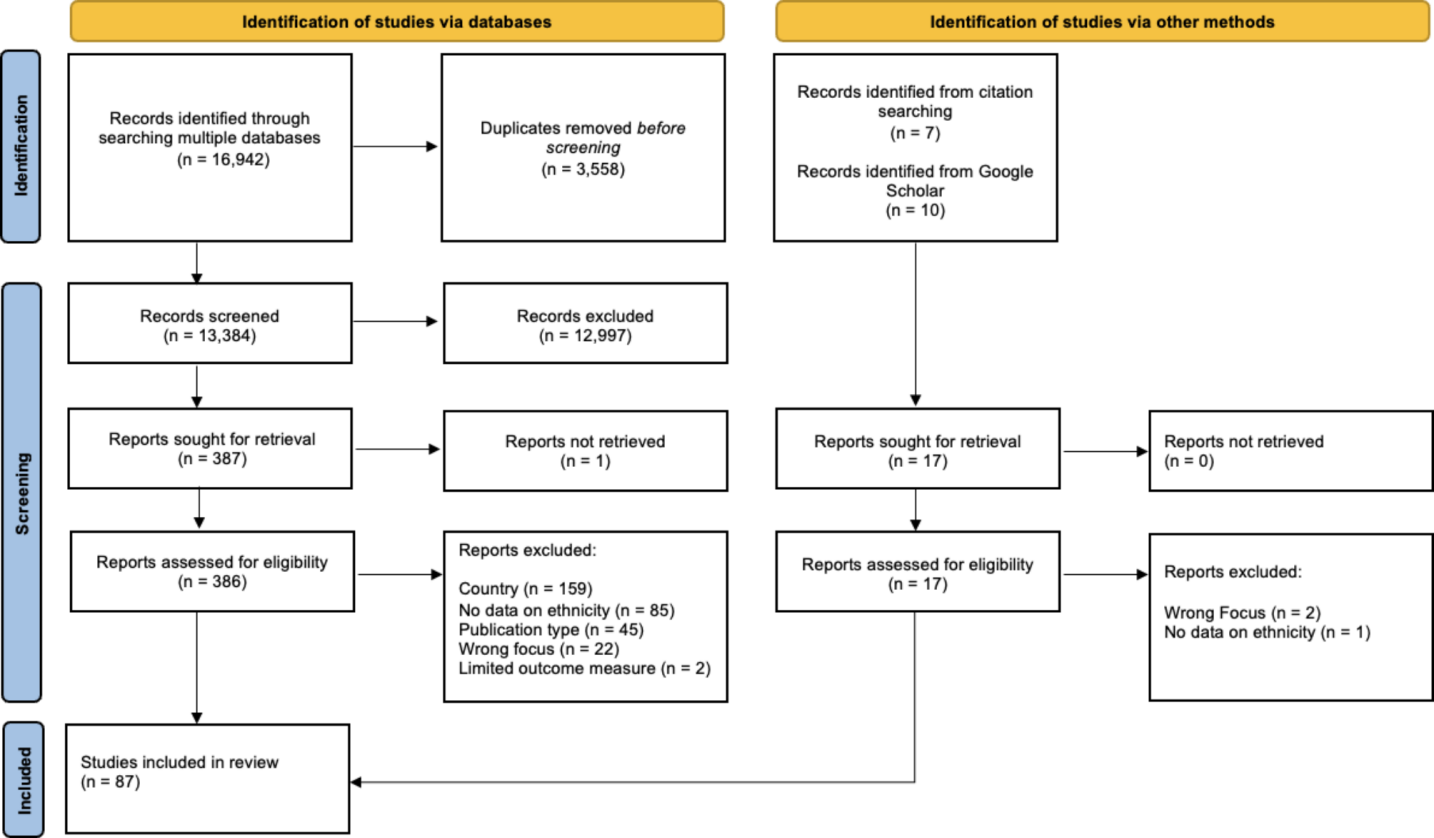
PRISMA flowchart

Database search results were imported into EndNote 21 [48] and duplicates were removed. Remaining articles were imported into Rayyan [49], which was used for screening. Article titles and abstracts were screened for relevance by two independent reviewers (RM, HT). Articles eligible for full text screening were sought and screened against eligibility criteria by two independent reviewers (AWR, HT). Five full text articles were sent to a third reviewer (SM) for arbitration of disagreements, four of which were excluded. Reference lists of included articles were also searched to identify any further relevant articles. See Fig. 1 for the PRISMA flow diagram. A list of excluded articles is available in Additional File 2.

### Data extraction and synthesis

Data were extracted by AWR using a Microsoft Excel data extraction form developed and piloted by HT and AWR. For each study, the author/s, year of publication, country of study, study aim, design and methods, sample size, participant characteristics (e.g., sex, age, ethnicity, religion, SES, co-occurring disorders), type of clinical ED or disordered eating behaviour/s examined, and relevant findings were extracted. Extracted data were synthesised by AWR. A descriptive numerical summary of study characteristics was performed, alongside a narrative summary of findings relating to prevalence, access to treatment and treatment experience.

The central focus/foci of each study were determined according to the primary aim/s reported by authors. Participant characteristics were recorded as reported in studies. When grouping studies according to sample age, where possible, age range was used over mean age as this provided more explicit information regarding the age composition of the sample. Clinical samples were those that recruited individuals with a diagnosed ED.

Given there is no international classification system for ethnicity, a custom set of ethnic categories were developed guided by those used by Egbert et al. [14] (see Table 2). The ‘unspecified other’ category refers to studies in which participants from several ethnic groups were collapsed into a single category (e.g., ‘other ethnicity’, ‘non-White’, ‘other Australian’). The Indigenous category reflects participants who represent the original inhabitants of a particular country (e.g., Aboriginal Australians, Torres Strait Islanders, Māori, First Nations and Métis Inuit).

**Table 2:** Number and percent of studies by participant demographics and study design

Sample/Study Characteristic	Country ^a^				Total (*N* = 87)
	Australia (*N* = 14)	Canada (*N* = 9)	Aotearoa New Zealand (*N* = 11)^f^	UK (*N* = 53)	
Sex					
Girls and Woman	8 (57%)	6 (67%)	2 (18%)	30 (57%)	**46 (53%)**
Boys and Men	0	1 (11%)	1 (9%)	1 (2%)	**3 (3%)**
Mixed Sex	5 (36%)	2 (22%)	8 (73%)	22 (42%)	**37 (43%)**
Not Reported	1 (7%)	0	0	1 (2%)	**2 (2%)**
Age					
Children & Adolescents (0–17 years)	3 (21%)	0	0	19 (36%)	**22 (25%)**
Adults (≥ 18 years)	5 (36%)	7 (78%)	4 (36%)	24 (45%)	**40 (46%)**
Mixed Age Range	6 (43%)	2 (22%)	7 (64%)	8 (15%)	**23 (26%)**
Not Reported	0	0	0	5 (9%)	**5 (6%)**
Ethnicity^d^					
Asian	10 (71%)	6 (67%)	5 (45%)	47 (89%)	**68 (79%)**
*Asian (east)*	5 (36%)	1 (11%)	2 (22%)	3 (6%)	**14 (16%)**
*Asian (south)*	0	1 (11%)	0	33 (62%)	**34 (40%)**
*Asian (unspecified)*	5 (36%)	4 (44%)	1 (9%)	11 (21%)	**21 (24%)**
Black	1 (7%)	2 (22%)	0	21 (40%)	**24 (28%)**
*Black (African)*	1 (7%)	1 (11%)	0	3 (6%)	**5 (6%)**
*Black (Caribbean)*	0	0	0	10 (19%)	**10 (12%)**
*Black (unspecified)*	0	1 (11%)	0	8 (15%)	**9 (10%)**
Hispanic	0	3 (33%)	0	0	**3 (3%)**
Indigenous	4 (29%)	5 (56%)	6 (55%)	0	**15 (17%)**
Pacific Islander	0	0	3 (27%)	0	**3 (3%)**
Multiracial/Multiethnic	0	0	1 (9%)	8 (15%)	**9 (10%)**
Middle Eastern	0	2 (22%)	1 (9%)	1 (2%)	**4 (5%)**
South American	0	0	1 (9%)	0	**1 (1%)**
White/European	7 (50%)	7 (78%)	3 (27%)	34 (64%)	**51 (59%)**
Unspecified other	7 (50%)	1 (11%)	3 (27%)	12 (23%)	**23 (27%)**
Not Reported	0	0	0	3 (6%)	**3 (3%)**
Religion					
Reported Religion	2 (14%)	2 (22%)	0	15 (28%)	**19 (22%)**
SES					
Reported SES	8 (57%)	3 (33%)	4 (36%)	20 (38%)	**35 (40%)**
Co-morbidity					
Reported Co-morbidity	0	0	6 (55%)	14 (26%)	**20 (23%)**
Study Design					
Case Study/Series	0	1 (11%)	1 (9%)	8 (15%)	**10 (11%)**
Quantitative	14 (100%)	7 (78%)	9 (82%)	36 (68%)	**66 (77%)**
Qualitative	0	1 (11%)	1 (9%)	8 (15%)	**10 (11%)**
Mixed Method	0	0	0	1 (2%)	**1 (1%)**
Sample Type					
Clinical Sample	1 (7%)	2 (22%)	3 (27%)	21 (40%)	**27 (31%)**
Non-Clinical Sample	12 (86%)	7 (78%)	7 (64%)	26 (49%)	**52 (60%)**
Clinicians	0	0	0	3 (6%)	**3 (3%)**
Mixed Sample^b^	1 (7%)	0	1 (9%)	3 (6%)	**5 (6%)**
EDs in Clinical Samples^b^					
AN	2 (100%)	2 (100%)	4 (100%)	14 (64%)	**22 (73%)**
BN	2 (100%)	1 (50%)	3 (75%)	12 (55%)	**17 (60%)**
AN & BN	0	0	1 (25%)	2 (9%)	**3 (10%)**
BED	0	1 (50%)	1 (25%)	1 (5%)	**3 (10%)**
EDNOS	2 (100%)	0	2 (50%)	5 (23%)	**9 (30%)**
OSFED	0	0	1 (25%)	1 (5%)	**2 (7%)**
Not Reported	0	0	0	3 (14%)	**3 (10%)**
Disordered Eating Measure					
EAT-26	6 (43%)	1 (11%)	2 (18%)	14 (29%)	**23 (26%)**
EAT-40	0	0	1 (9%)	1 (2%)	**2 (2%)**
EDI	0	4 (44%)	2 (18%)	0	**6 (7%)**
EDI-2	3 (21%)	1 (11%)	0	0	**4 (5%)**
EDI-3	0	0	0	1 (2%)	**1 (1%)**
EDI-SC	3 (21%)	0	0	0	**3 (3%)**
EDE	4 (29%)	0	0	2 (4%)	**6 (7%)**
EDE-Q	2 (14%)	0	1 (9%)	1 (2%)	**4 (5%)**
EDE-S	1 (7%)	0	0	0	**1 (1%)**
ED-15	0	0	0	1 (2%)	**1 (1%)**
SEED	0	0	1 (9%)	0	**1 (1%)**
SCOFF	0	0	0	3 (6%)	**3 (3%)**
BITE	0	0	0	3 (6%)	**3 (3%)**
BULIT-R	0	2 (22%)	0	0	**2 (2%)**
BEQ	0	0	0	1 (2%)	**1 (1%)**
DEBQ	0	0	0	5 (10%)	**5 (6%)**
NEQ	1 (7%)	0	0	0	**1 (1%)**

^a^. Some categories are not mutually exclusive, and percentages are rounded, as such totals may not add up to 100

^b^. Mixed sample refers to studies which recruited multiple stakeholder types in their sample (e.g., clinicians, family members, community members, individuals with an eating disorders)

^c^. The number of studies which included participants diagnosed with an eating disorder (*N* = 30) was used to calculate percentages rather the overall number of studies

^d^. Figures represent the number and percent of studies which included participants of this particular ethnic group

^e^ Three included papers from New Zealand were from the same study – the NZMHS. We included them as separate studies since each paper has been governed by different research questions and different analyses

Abbreviations: AN – Anorexia Nervosa; BN – Bulimia Nervosa; BED – Binge Eating Disorder; EDNOS – Eating Disorder Not Otherwise Specified; OSFED – Other Specified Feeding or Eating Disorder; EAT-26, EAT-40 – Eating Attitudes Test; ED-15 – Eating Disorder-15; EDI – Eating Disorder Inventory; EDI-SC – Eating Disorder Inventory Symptom Checklist, EDE – Eating Disorder Examination; EDE-S – Eating Disorder Examination Screening Version; EDE-Q – Eating Disorder Examination Questionnaire; SEED – Short Evaluation of Eating Disorders; BITE – Bulimic Inventory Test, Edinburgh; BULIT-R – Bulimia Test Revised; BEQ – Binge Eating Questionnaire; DEBQ – Dutch Eating Behaviour Questionnaire; NEQ – Night Eating Questionnaire; SES – Socioeconomic Status

The study team met regularly throughout the data extraction and synthesis process to discuss progress and resolve any queries around the grouping and classification of studies. A second author (HT) also independently verified the categorisation of a randomly selected subset of 10% of included studies. In line with guidance on conducting scoping reviews, critical appraisal of the included studies was not conducted [42, 43].

## Results

Overall, the search strategy identified 16,942 articles, of which 3,558 were duplicates. Titles and abstracts of the remaining 13,384 articles were screened resulting in the exclusion of 12,977 articles. The full text of the remaining 387 articles were sought. It was not possible to retrieve the full text for one article [50]. After examining the other 386 articles against eligibility criteria, 313 articles were excluded, leaving 73 articles for inclusion. A further nine and five articles were identified through Google Scholar and hand searching of reference lists respectively. 87 articles were therefore included in this review, with 76 being journal articles and 11 theses. The study and sample characteristics of included studies are outlined in Additional File 3, with a summary presented in Table 2.

**Fig. 2:**
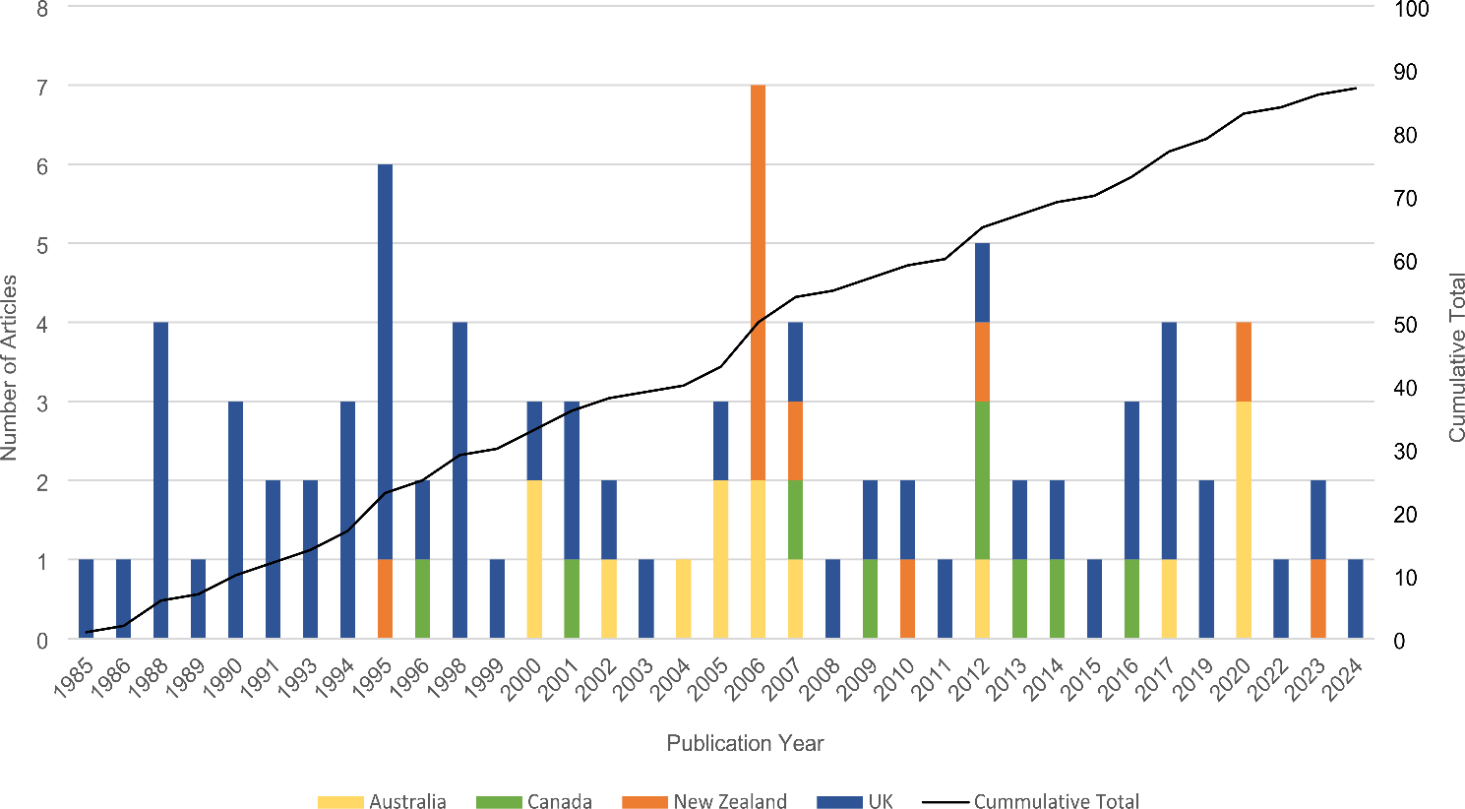
The number of included journal articles and theses by publication year and country

Most studies were conducted in the UK (61%, 53 studies), followed by Australia (16%, 14 studies), Aotearoa New Zealand (13%, 11 studies) and Canada (10%, nine studies). Included journal articles were published between 1985 and 2024 and unpublished theses completed between 1996 and 2017. The number of publications per year for each country, alongside the cumulative total of publications, is shown in Fig. 2.

### Study foci

**Fig. 3:**
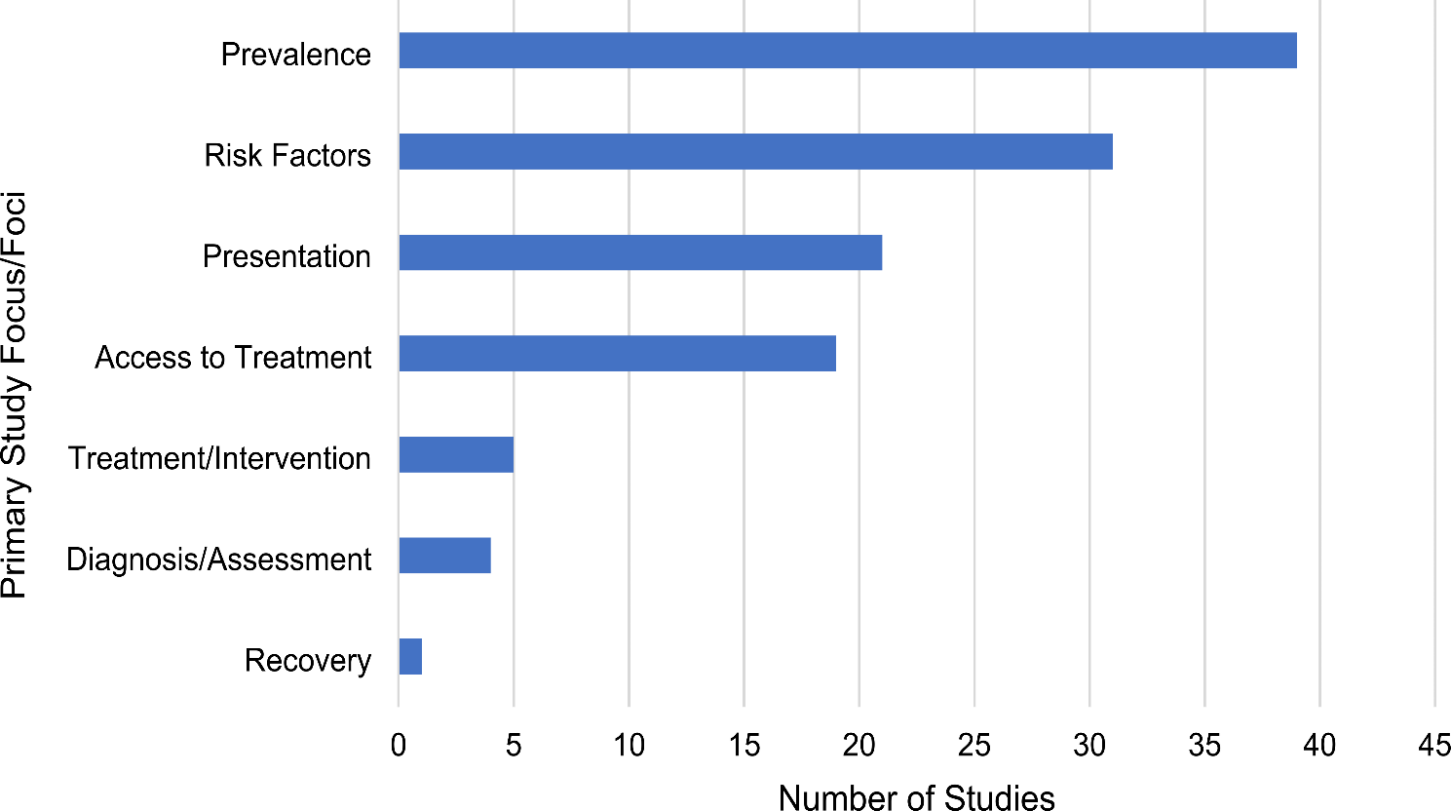
Primary focus/Foci of studies

Identified studies were heterogenous in their research aims, and the foci of studies varied greatly. Figure 3 shows studies categorised according to their central focus/foci. Topics most commonly addressed included prevalence, risk factors, clinical presentation and access to treatment. Risk factors studied included acculturation, ethnic identity, cultural conflict, parental overprotection, body image dissatisfaction, self-esteem, religion, perfectionism, discrimination, abuse and trauma. Many studies also tangentially reported on areas outside of their primary focus and as such contribute findings to several research areas.

### Study design

Four broad categories of study design were identified: case studies/series (11%, 10 studies), qualitative studies (11%, 10 studies), quantitative studies (77%, 66 studies) and mixed-method studies (1%, one study).The vast majority of studies were cross-sectional surveys. Further details regarding study methods and findings are presented in Additional File 3.

### Measures of disordered eating and eating disorders

17 different validated interview and self-report measures were used to assess disordered eating behaviours (see Table 2). The most cited measure was the Eating Attitudes Test (EAT-26), used in 23 studies (26%). Studies assessing the prevalence of EDs in non-clinical samples (14%, 12 studies) used varying diagnostic criteria, including Russell 1979 criteria [86], DSM, Third Edition, Revised (DSM-III-R) [87, 107, 113, 117], DSM-IV [76–78], DSM-5 [10, 62–64] and International Classification of Diseases, Eleventh Revision (ICD-11) [63].

### Sample characteristics

Most studies were conducted with non-clinical samples (60%, 52 studies), including university students, schoolchildren or people drawn from the general population. 27 studies (31%) had clinical samples, three (3%) recruited only clinicians [119, 129, 134] and five (6%) included multiple participant types (e.g., community members, clinicians, ED patients, relatives) [59, 84, 124, 127, 130]. Samples including individuals with a diagnosed ED (30 studies, 35%) tended to encompass multiple ED diagnoses (15 studies), most commonly AN and BN. Three studies did not report the specific ED diagnosis of participants [98, 131, 135].

Sample size varied across studies. There were six case studies of a single participant [67, 74, 90, 91, 98, 102] and four case series, all with five or less participants [85, 89, 92, 95]. Across quantitative studies, sample size ranged from 14 [52] to 1,024,958 [135]. For qualitative studies, sample size ranged from one [133] to 44 [130] participants. More than half of included studies focused exclusively on girls and/or women (53%, 46 studies). 37 studies (43%) had mixed-sex samples and three (3%) studies had samples consisting of only boys or men, two of which were case studies [74, 102]. Participant ages ranged from 6 years [98] to 90 years [10, 126] and most studies focused on adult populations (46%, 40 studies). No studies focused specifically on middle age or older adults and only two studies focused specifically on children (≤ 12 years) [98, 104].

59 studies (68%) included participants from multiple ethnic groups. Of these studies, 38 had samples which were predominantly (i.e., > 50% participants) White. 25 studies (29%) focused exclusively on minority ethnic individuals, with over half of these (16 studies) specifically targeting Asian populations. Two qualitative studies did not report ethnicity of all participants [124, 130] and Currin et al. [119] did not report the ethnicity of clinicians recruited but vignette ethnicity was manipulated.

Across the reviewed studies, individuals of Asian ethnicity/origin were identified as the minority ethnic group most frequently included in studies (78%, 68 studies). In the UK, comparatively, there was a noticeable absence of research focusing on Black and multiracial individuals. There appeared to be a small but growing body of evidence regarding Aboriginal Australians and Torres Strait Islanders in Australia and Māori and Pacific peoples in Aotearoa New Zealand. Participant ethnicity was determined in several ways, most frequently through self-report (51%, 44 studies). Other studies determined ethnicity through country of birth, family ancestry or visual appearance^1^. 24 studies (28%) did not report how participant ethnicity was defined.

Participant religion was reported by 19 studies (22%). Muslim was the most cited religion (16 studies), followed by Hindu (nine studies), Christian (eight studies) and Sikh (seven studies). SES was reported in 35 studies (40%). Measures of SES varied greatly, making it difficult to compare and evaluate SES across studies. Co-occurring disorders were reported in 20 studies (23%). Most recorded co-occurring psychiatric conditions (20 studies), most commonly mood and anxiety disorders. Co-occurring physical conditions in comparison were only reported in three studies [91, 98, 136]. The rate of co-occurring disorders by ethnicity could not be determined for all studies [10, 76, 82, 116, 126, 136].

### Prevalence

57 studies (66%) reported findings relating to prevalence. The majority (38 studies) reported on the prevalence of disordered eating behaviours or individual ED symptoms (e.g., binge eating, purging, dietary restraint), whilst the rest focused on prevalence of clinical EDs. Prevalence was most commonly assessed with small scale cross-sectional surveys using convenience samples. Only a few large-scale population or community surveys/studies [10, 60–64, 76–78, 126] and two-phase prevalence studies [10, 86, 87, 107, 113, 117] were identified. Seven studies analysing patient records also reported prevalence related findings [83, 88, 118, 121, 122, 132, 135], across these studies there was a trend whereby minority ethnic patients in the UK and Māori patients in Aotearoa New Zealand were less likely to be diagnosed with AN but more likely to be diagnosed with BN [83, 88, 118, 121, 122, 132].

Prevalence estimates varied across studies, with inconsistent findings reported within and across ethnic groups. When compared to their White counterparts, Asian individuals in the UK generally had greater levels of disordered eating [87, 93, 96, 100, 104, 105, 114–116, 126], in particular bulimic behaviours and BN [87, 99]. For restrictive eating, the opposite pattern was more commonly found, with several studies reporting greater levels of dietary restraint in White girls and women [94, 97, 110]. Studies in Australia [55, 57, 58], Canada [66] and Aotearoa New Zealand [80] also indicated greater disordered eating in Asian adults and adolescents compared to other ethnic groups, including White. Though in some cases, differences were only found when using specific measures of disordered eating [57, 58].

A small number of studies also found Indigenous groups to have higher levels of disordered eating and EDs compared to other ethnic groups. Findings from the 2006 New Zealand Mental Health Survey (NZMHS) highlighted higher prevalence of DSM-IV EDs, namely AN and BN, for Māori [76, 77]. In Australia, greater prevalence of both disordered eating [60] and DSM-5 EDs [63, 64], in particular Unspecified Feeding or Eating Disorder [64] and OSFED-Night Eating Syndrome [63] were found for Aboriginal and Torres Strait Islander adults and adolescents. Further, in a Canadian study, Aboriginal women exhibited greater levels of bulimic behaviours compared to White women [65].

Prevalence data for other minority ethnic groups was limited. However, higher prevalence of DSM-IV AN and BN was also found for Pacific peoples in the NZMHS [76, 78]. There was also some evidence of higher rates of bulimic symptoms and/or BN in Black individuals in the UK [10, 107] and Hispanic adults in Canada [68]. In addition, particularly high levels of disordered eating behaviours were reported for ‘mixed race’ [116] and ‘mixed/other’ [126] individuals in the UK. Notably, across all four countries several studies found few or no ethnic differences in disordered eating [41, 51, 56, 69, 71, 79, 101, 109, 111–113, 117, 125] or rates of diagnosable EDs [10].

### Access to treatment

Findings relating to access to ED treatment for minority ethnic individuals were reported in 25 studies (29%). Most studies reporting access related findings were conducted in the UK (20 studies) and focused exclusively on South Asian populations (10 studies).

#### Referral rates and service utilisation

In the UK, lower referral rates were found for Asian and Black individuals, when compared to their White counterparts [88, 89, 95, 103, 108, 121, 122]. In some instances, ethnic differences in referral rates were greater than expected based upon the local catchment area and/or known prevalence estimates [89, 108, 121, 122]. Similarly, analysis of a national dataset from Aotearoa New Zealand found rate of presentation to specialist mental health services for Māori with EDs was lower than expected [83]. Data from the NZMHS also highlighted that for Pacific people rate of 12-month mental health service utilization was lowest for EDs compared to other psychiatric conditions [78].

In the UK, ethnic disparities in referral routes were also found. One case note review comparing White and Black ED patients found Black patients were more frequently referred from emergency psychiatric services than primary care services [88]. A vignette study identified a similar trend whereby general practitioners (GPs) were more likely to offer African-Caribbean patients a follow-up appointment rather than a direct referral to secondary mental health services [119]. Data on the ethnicity of ED referrals in Australia and Canada were unavailable.

Post-referral, the type of treatment received by patients did not differ significantly between ethnic groups in the UK [121, 122], nor between Māori and non-Māori in Aotearoa New Zealand [83]. Reflective of these findings, in vignette studies from the UK, ethnicity had no significant impact on the treatment recommendations selected by clinicians [119, 129]. Non-significant trends, however, indicated that for restrictive eating presentations, South Asian cases were offered high intensity cognitive behavioural therapy (CBT) less frequently than White cases [129] and South Asian women patients with BN were less likely to receive interpersonal psychotherapy (IPT) compared to non-Asian patients [121]. Poorer attendance to motivational groups for Māori ED patients [82] and virtually delivered guided self-help for minority ethnic patients with bulimic spectrum EDs in the UK [136] was also reported.

#### Barriers to help-seeking and accessing treatment

Various barriers to help-seeking and access to treatment were identified across 10 qualitative studies [73, 84, 120, 123, 124, 127, 131, 133, 134] and one case study [102]; these are summarised in Table 3.

**Table 3:** Barriers to help-seeking and accessing treatment

Barrier Identified	Number of Studies (*n* = 11)
Stigma and shame around mental health and EDs	10
Lack of knowledge around EDs in minority ethnic populations	10
Poor family support	6
Fear of mental health services/psychological intervention	5
Differences in communication of symptoms	5
Past negative healthcare experiences	4
Systemic/service barriers	3

Abbreviations – ED – eating disorder

Shame and stigma and lack of knowledge were the most commonly cited barriers.

Several UK qualitative studies of minority ethnic groups, mainly South Asian [120, 123, 124, 127, 130, 131, 133, 134], and one qualitative study of South Asian women in Canada [73], found high levels of stigma around EDs and mental health result in shame and secrecy which subsequently delay help-seeking. Individuals often refrained from disclosing eating or mental health difficulties for fear of being ostracised or excluded by their family or the wider community [73, 120, 123, 124, 127, 134]. Additionally, in some South Asian communities, cultural denial of mental health issues and belief in resolving difficulties alone seemed to further reinforce this silence [73, 130, 133]. Māori ED patients also discussed stigma in relation to a hierarchy of EDs, with AN being perceived as more serious than BN and BED, and thus more worthy of treatment [84].

Lack of knowledge about EDs and their severity was thought to delay identification and help-seeking for minority ethnic groups, especially South Asian [73, 120, 123, 124, 127, 130, 131, 133]. This issue was particularly apparent across older generations [123, 124, 127, 130], with younger individuals having greater mental health literacy through teaching at school and media exposure [123, 124]. Other studies recognised lack of clinician knowledge about EDs in minority ethnic individuals in the UK [120, 124, 127, 131, 134] and Māori in Aotearoa New Zealand [84], may also prevent access to treatment.

Other barriers identified included scepticism towards mental health services [73, 102, 123, 130, 133] and lack of familial support, with parents often failing to acknowledge and accept the ED [73, 120, 123, 127, 133, 134]. Differences in how ED symptoms are communicated and conceptualised were also thought to pose difficulties for identification and thus access to treatment. For some minority ethnic patients, there was a tendency to discuss their eating difficulties from a physical health perspective [102, 124, 127, 130, 134], often minimising shape and weight concerns [134]. Religious and spiritual beliefs also influenced interpretation of EDs, for example, some individuals understanding the ED as being the result of a possession [102, 134].

Previous negative experiences with healthcare services also delayed help-seeking. Such incidents often occurred in primary care settings, with GPs failing to identify EDs or take concerns of Māori [84] or Black and South Asian patients seriously [124, 127]. South Asian patients in particular, expressed apprehension regarding privacy and confidentiality [123, 127, 130]. Other systemic and service-related barriers included limited resources (e.g., number of inpatient beds) [84], inaccessible service locations [84], service threshold criteria [134], and use of inconsistent, inadequate and Eurocentric assessment methods [84, 127]. Facilitators of accessing treatment for Māori were reported by Clark et al. [84]; these included self-advocacy, positive support systems (e.g., friends, family, clinicians), ED health literacy and promotion in media.

### Treatment experience

Few studies specifically explored the treatment experiences of minority ethnic patients. Birmingham and Sidhu [67] describe the case of a Canadian Southeast Asian woman with AN who chose zen therapy over conventional treatment. Seven other UK case studies and series briefly touched on treatment experiences [89–92, 95, 98, 102]. Experiences of treatment were also mentioned in five qualitative studies from the UK from the perspectives of clinicians [124, 127, 134], patients [123, 127, 131] and their relatives [124, 127]. However, only two of these studies [123, 134] set out to examine treatment experiences. Of the 13 studies (15%) reporting findings related to treatment experience, all but one [67] was conducted in the UK and most focused exclusively on Asian patients (8 studies).

Across studies, reference was made to the use of individual, group and family therapy in both in-patient and out-patient settings, though the exact content of interventions was often unclear. Bryant-Waugh and Lask [95] however, noted that addressing family issues and cross-cultural conflict was often a central focus of intervention for South Asian adolescents with AN in the UK.

Several UK studies highlighted difficulties in engaging Asian patients and their families in ED treatment [91, 92, 95, 98, 123], particularly with family therapy [92, 95, 123]. This in some cases led to premature discharge and termination of treatment [92, 95]. Hoque [123] attributed poor engagement among South Asian families to an interdependent sense of self and parental apprehension towards conventional treatment [123]. Preferences for culturally familiar treatment approaches including religious clergy and alternative medicine were also noted in a case study of a Southeast Asian woman with AN in Canada [67] and a Chinese boy with AN in the UK [102]. In another UK qualitative study, reluctance of minority ethnic patients to share details about their ED with their parents was also thought to complicate family approaches to treatment [131]. Some relatives of minority ethnic individuals in the UK also reported experiences of inadequate treatment, including delayed or misdiagnoses, negative cultural stereotypes held by clinicians and lack of effective and sensitive communication within services [124].

In one UK study, minority ethnic patients raised concerns over the use of Eurocentric interventions and felt culture and race were inadequately and inconsistently addressed within treatment [131]. Likewise, some clinicians questioned the utility and appropriateness of current National Institute for Health and Care Excellence [137] recommended treatments [134]. For example, the observation that for minority ethnic individuals ED symptoms may be driven by affective and relational factors, over and above shape and weight concerns, led to uncertainty around the use of treatment models such as CBT which place emphasis on identifying and challenging beliefs around food, body image and weight [134]. Clinicians communicated a desire to adapt evidence-based interventions to make them culturally sensitive and specific but felt restricted in doing so by service structures and guidelines and a lack of time, resources, confidence and knowledge [124, 127, 134].

ED treatment was, however, also referred to positively. In one UK study, South Asian girls and women emphasised the importance of their ED being acknowledged by clinicians during the treatment process [123]. The validating and supportive response of clinicians was directly contrasted against that of parents, who often refused to accept or recognize their daughter’s eating difficulties [123]. Developing a sense of self outside of the family and beginning to identify, understand and meet their individual needs was also considered an important part of the recovery process [123].

## Discussion

This scoping review aimed to explore literature on disordered eating and EDs in minority ethnic individuals in Australia, Canada, Aotearoa New Zealand and the UK, with a special focus placed on prevalence, access to treatment and treatment experience, given their relevance in better understanding the treatment needs of minority ethnic populations. The review reveals significant differences across the four countries, reflecting varying levels of attention to equity and unique systemic challenges. Across all countries, studies highlighted systemic inequities in ED recognition, diagnosis, and treatment, including disparities in referral rates, access to services, and culturally sensitive care. In the following, the main findings are discussed in relation to the wider literature, clinical practice and relevant policies and guidelines. Based on identified gaps and/or limitations in the literature, recommendations for future research are then made.

### Study and sample characteristics

Approximately 60% of identified studies were conducted in the UK which reflects the country’s longer history of addressing EDs in ethnic minorities and focusing on health inequities, though structural inequalities persist [138, 139]. The unequal distribution of studies across countries may also reflect national variation in research institutions’ priorities and funding, but also systemic inequities in how race and ethnicity data are collected and utilised within healthcare systems [14, 140]. For example, Australia and Canada have been criticised for their inadequate collection of such data, which is a fundamental barrier to understanding and addressing disparities in ED prevalence and treatment access [140–142]. Data collection may be more difficult among Indigenous populations in Australia and Canada, many of whom live in remote regions, however, the lack of consistent data collection systems perpetuates inequities by obscuring the needs of minority ethnic populations, hindering the development of equitable healthcare policies and services.

The primary foci of included studies were diverse and broadly similar to those of US studies [16]. However, there was a notable lack of research exploring the assessment, diagnosis and treatment of EDs in minority ethnic individuals. This gap likely reflects systemic barriers that contribute to the underrepresentation of these groups in clinical settings [18, 143, 144], including institutional racism, culturally insensitive practices, and historical mistrust of medical research within marginalized communities [145–147]. These barriers are exacerbated by socio-political factors, such as histories of colonisation and ongoing discrimination, which shape healthcare experiences and access for both immigrant and Indigenous populations [149].

Most studies used cross-sectional and survey designs, which, while cost-effective, have notable limitations, such as the inability to establish causality and the frequent use of measures not validated for use in minority ethnic populations [150, 151]. Commonly used tools, like the EAT-26, may not adequately capture the nuances of disordered eating [151] in these groups, as, for example, cultural or religious reasons can lead to misinterpretation of items [150]. Conflicting findings from studies assessing the cross-cultural validity of the EAT-26 [80, 96] highlight the need for diagnostic tools that are rigorously validated for diverse and representative samples.

Individuals of Asian ethnicity/origin were included in studies more often than any other minority ethnic group. This in part may be due to most reviewed studies being conducted in the UK, where Asian, particularly South Asian, form the largest minority group (9.3%) [152]. Australia, Canada and Aotearoa New Zealand also have substantial Asian populations, including from East Asia, due to historical and contemporary immigration patterns [153–155]. Unless studies exclusively recruited minority ethnic participants, samples tended to be predominantly White. Challenges in recruiting minority ethnic individuals into research include language and cultural barriers, mistrust of research, concerns about potential negative outcomes, and competing logistical demands – issues also noted in other healthcare contexts [148, 156–158]. These challenges sometimes preclude statistical analysis of minority ethnic groups or lead to multiple minority ethnic groups being collapsed into a single category to increase statistical power. This risks obscuring critical differences between groups, such as those between immigrant minority populations and Indigenous peoples whose experiences are uniquely shaped by colonization. Such practice also compromises the specificity and generalizability of results.

The measurement and reporting of ethnicity across studies varied and was inconsistent. This was perhaps to be expected given that at present there is no globally recognised definition of ethnicity, and its conceptualisation and categorisation can vary dependent upon cultural context [14, 159, 160]. For example, in Australia it is common practice for researchers to only collect data on variables which measure cultural and language diversity (i.e., country of birth, language spoken). Though culture and language are important determinants of access to and experiences of treatment, such practice can lead to the aggregation of several ethnic groups into a single category and may overlook important factors such as generational status, acculturation or being a visible racial minority [142].

Terminology to describe the ethnicity of samples was also inconsistent. As noted by Cummins et al. [161], the same label was applied to samples which differed considerably with regards to ethnicity, culture and country of origin. For example, most likely stemming from differing immigration patterns, ‘Asian’ usually referred to those of South Asian descent in UK studies but to those of Southeast or East Asian descent in Australian, Canadian or Aotearoa New Zealand studies.

### Prevalence

In line with findings from the US, prevalence estimates varied both within and across ethnic groups, depending on the type of disordered eating behaviour or ED being studied [16]. Variability in study design, diagnostic criteria or measures used, and population studied, likely also contributed to inconsistent findings and made synthesis and interpretation of prevalence estimates challenging [10].

Across all four countries, there was a lack of large-scale population-based or epidemiological studies assessing prevalence. Those identified were often flawed, for example, by not assessing all types of ED symptoms [60, 61] or diagnoses [76–78], lack of co-production [64] and low numbers of minority ethnic individuals within the sample [60, 61, 63]. This reflects a broader lack of prioritisation of minority health within national research agendas, perpetuating inequities in health knowledge and study design. Comparatively, in the US, several large-scale studies have provided prevalence estimates for ED diagnoses across varying ethnic groups [12, 162, 163].

Use of Eurocentric diagnostic criteria or culturally inappropriate diagnostic measures [9] highlights how systemic inequities shape the identification and diagnosis of EDs, disadvantaging minority ethnic populations and likely underestimating the true burden of EDs in these groups. Further complicating the interpretation of prevalence estimates, differences in the operationalisation of ethnicity [114] and individual and sociodemographic characteristics including age [63], BMI [62, 97], religious affiliation [100], psychosocial functioning and mental health [63, 64] were found to influence and explain ethnic differences in reported rates of disordered eating and EDs.

### Access to treatment

Consistent with findings from US studies [18], poorer access to treatment was found for Black and Asian individuals in the UK [88, 89, 95, 103, 108, 121, 122] and Māori and Pacific people in Aotearoa New Zealand [78, 83]. Ethnicity data for referrals to services in Australia and Canada were not available. Contrary to early ideas [108], prevalence data suggests that lower referral rates for minority ethnic and Indigenous individuals are unlikely to be a consequence of reduced need for ED treatment. Instead, there seems to be a number of individual, cultural and systemic barriers to help-seeking and accessing ED treatment, reflecting deeply embedded inequities in healthcare systems. Some identified barriers such as shame, stigma and lack of clinician knowledge appear to be common across all individuals affected by EDs, regardless of ethnicity [164, 165]. However, the way in which they manifest may qualitatively differ across ethnic groups. For example, in South Asian communities, stigma appears to be rooted in and perpetuated by cultural and religious beliefs around mental health [73, 130, 133], whilst barriers such as cultural stereotypes held by clinicians appear unique to minority ethnic and Indigenous groups [84, 124, 134]. Of note, unlike in US studies, cost of treatment was not cited as a barrier [166, 167], reflecting differences in the healthcare systems of the countries studied.

Ethnic differences in the type of ED treatment received were minimal [83, 119, 121, 122, 129]. Waller et al. [121] suggest this may indicate that after initially accessing services health inequities are reduced. However, as with most of the identified studies, data on treatment attendance and outcomes were not reported, suggesting their conclusion may be premature. Further, there were trends for minority ethnic patients to be offered different types and intensities of treatment [119, 122, 129], suggesting inequities may persist even after initial service access.

### Treatment experience

Treatment experience was understudied across all countries. The limited data available from UK and Canadian studies focused largely on the experiences of Asian individuals and often lacked detail and/or had limited generalisability due to small and idiographic samples.

Reviewed studies highlight that for some minority ethnic communities there may be culturally specific understandings of eating difficulties and a preference for culturally sanctioned treatments, especially among older generations. The poor treatment engagement observed in some Asian families [91, 92, 95, 98, 123], may therefore to some extent be attributable to a disconnect between these individuals’ cultural beliefs and preferences and conventional ED treatment modalities. For example, the appropriateness of current evidence-based ED treatment for minority ethnic patients was questioned by both clinicians [134] and patients [131] in the UK. This points to a systemic failure to integrate equity into service design, overlooking culturally appropriate practices in favour of Eurocentric approaches which may not resonate with, or be accessible to, minority ethnic patients.

Engagement challenges and poor treatment experiences are not necessarily unique to minority ethnic groups. General challenges, including managing ambivalence and lack of motivation to change, may be faced when working with any patient [168], and any individual, regardless of their identity, may have negative treatment experiences. However, the latter may be more common among those who hold a minoritised or marginalized identity [8]. This may reflect structural inequities, such as a lack of workforce diversity in ED services and an absence of culturally sensitive therapeutic approaches [169].

Positive aspects of treatment, including feeling listened to, supported and understood, were discussed by South Asian women in one UK study [123]. This may reflect an essential aspect of therapy, the need for a strong therapeutic alliance [170]. Further research is needed to elucidate any culture or ethnicity specific factors which contribute to positive treatment experiences, for example, specific cultural adaptations [169, 171].

### Future research

This review highlights the ongoing need for research into disordered eating and EDs among minority ethnic individuals in Australia, Canada, Aotearoa New Zealand and the UK, which currently lag behind the US in terms of research in this area.

Standardising the collection and reporting of race and ethnicity data across countries is critical to promoting equity in ED research and treatment [14]. Researchers should be transparent about their methods for reporting and collecting race and ethnicity data and avoid treating minority ethnic groups as homogenous populations. Broad, non-specific, ethnic groupings (e.g., Asian) and collective terminologies (e.g., non-White) should be avoided [172], with efforts made to target individual minority ethnic groups to allow for a deeper understanding of EDs within specific populations [173].

In addition to self-reported race or ethnicity, researchers should consider related factors, such as country of birth, language spoken, generational status, acculturation, religion and SES. These elements, along with socio-political determinants such as colonial histories and systemic racism, can provide a deeper understanding of how ethnicity influences ED prevalence, recognition and treatment disparities. Some scholars, however, argue that assessing ethnic differences in disordered eating and EDs is overly simplistic [111] and instead suggest focusing on individual cultural values and beliefs [111], for example, those around gender roles, appearance, family relationships, materialism, achievement, food and diet.

Future research must ensure samples are diverse in terms of age and sex, with increased inclusion of boys and men, children and middle to older adults. Attempts to address intersectionaI identities should also be made, irrespective of more complex study designs and analyses [174]. Emerging evidence in the US suggests increased risk of EDs in those who hold multiple marginalized identities [175–177].

Accurate prevalence estimates for specific ED diagnoses by ethnicity are essential to understand healthcare needs and inform service planning [63, 178]. Large scale epidemiological studies may be best to establish such estimates [178]. However, to ensure sufficient numbers of minority ethnic individuals to permit statistical analysis, oversampling of minority ethnic groups, as done in the NZMHS for Māori and Pacific peoples [76–78], may be required. Consideration of the appropriateness and validity of the chosen diagnostic measure for the population being studied is also important, and further development of culturally sensitive, non-Eurocentric diagnostic criteria and assessment measures may be required.

Indigenous populations may be hesitant to engage with Western research due to historical practices of research being conducted “on” or “to” them, rather than “with” them [179, 180]. To improve participation in research, there is a need for co-production and the active involvement of minority ethnic individuals throughout all stages of research [181, 182]. Decolonizing research and academic spaces is an essential part of this process, requiring the dismantling of Eurocentric frameworks that marginalize non-Western perspectives and the active inclusion of Indigenous knowledge systems and methodologies [183–185]. This approach can help ensure research is both culturally appropriate and empowering for historically marginalized groups. In Australia and Canada where the large land masses encompass many Indigenous groups with diverse languages and cultural variations, careful co-design and support for Indigenous researchers are paramount, as well as time to build trust among Indigenous groups [186].

Barriers to accessing ED treatment for minority ethnic and Indigenous groups require further exploration, as does understanding how treatment experiences and outcomes vary by ethnicity. Randomised controlled trials (RCTs) conducted in the US have examined ethnic differences in ED treatment outcomes [187–190] but similar studies are needed outside of the US to assess the feasibility, acceptability and effectiveness of existing and culturally adapted interventions across ethnic groups. Qualitative research with patients, relatives and clinicians can provide insight into the acceptability and experiences of interventions, including reasons for treatment drop out or decline. Identifying effective treatment models and culturally sensitive strategies will support the development of services that better meet the needs of diverse populations.

### Implications for clinical practice

Access to ED treatment for minority ethnic groups remains inadequate, with shame, stigma and a lack of knowledge cited as primary barriers. Addressing inequities in ED care requires systemic changes in how services are delivered and accessed. Public health campaigns should prioritise engaging minority ethnic communities in culturally relevant ways, acknowledging and addressing the historical and ongoing inequities that contribute to mistrust and underutilisation of services. Targeted media campaigns and advertisement within local communities, clubs, schools and places of worship could help to reduce stigma and promote help-seeking in relation to EDs [73, 127, 130, 134]. Training for healthcare professionals, especially those working in primary care, is also recommended to increase clinician confidence and competence in recognising and addressing EDs in minority ethnic and Indigenous individuals [73, 84, 120, 124, 127, 130, 134]. These training programmes and ED services could be co-produced with individuals with lived experience. To recruit minority ethnic individuals to such co-production initiatives, services may also need to enhance their appeal to minority ethnic individuals, for example, by using promotional materials featuring patients from a diverse range of backgrounds. Additionally, workforce diversity is essential to dismantling implicit biases and fostering equitable care environments.

Based on the scope of this review, and the identified evidence being limited in quality and quantity, it is not possible to conclude whether particular ED treatments are feasible or acceptable for minority ethnic patients. However, findings suggest that existing ED treatments, including family therapy and psychological and interpersonal therapies, may require further evaluation for minority ethnic groups. At present, although existing ED treatment guidelines (e.g., The Royal Australian and New Zealand College of Psychiatrists (RANZCP) and NICE ED guidelines) [137, 191] and professional bodies (e.g., National Eating Disorder Collaboration, NEDC) advocate for and recommend culturally responsive assessment and treatment, there are a lack of specific and clear recommendations for working with individuals from diverse cultural and ethnic backgrounds. Moreover, guidance should also recommend addressing structural barriers to access and ensuring minority ethnic patients’ voices are central to service design and evaluation.

### Strengths and limitations

Input and consultation from an academic librarian and clinical psychologist working in the ED field was sought during various stages of the review. No date limits were applied to searches, and broad search terms were used. Theses were included, minimising the risk of publication bias. However, a specific search for grey literature was not conducted meaning other relevant unpublished papers may have been missed.

Quality assessment of included studies was not conducted. This is in line with recommendations for conducting scoping reviews [42, 43] and given the heterogeneity of included studies, assessing quality would likely have proven challenging. The review only included studies from Australia, Canada, Aotearoa New Zealand and the UK, potentially introducing selection bias. We acknowledge EDs are not limited to Western or high-income countries [7] and other countries also have established minority ethnic populations. However, extending inclusion to US and other countries would not have been feasible given time and resource constraints. Studies not published in English were excluded, possibly leading to further selection bias.

During the data synthesis stage, studies were categorised using pre-determined categories designed by the research team. Due to resource constraints, this was done only by a single author (AWR). Despite measures to ensure this process was systematic and transparent, including spot-checking, regular study team meetings and documentation of decisions regarding categorisation, it is acknowledged some degree of subjectivity remains in this process. We were unable to conduct a thematic synthesis of treatment experience findings (see protocol [44]) due to a limited number of qualitative studies in this area.

## Conclusion

This scoping review provides a detailed overview of research into disordered eating and EDs in minority ethnic individuals in Australia, Canada, Aotearoa New Zealand and the UK, shedding light on systemic inequities that influence ED prevalence, recognition, and treatment in these populations. Addressing these disparities requires a paradigm shift toward equity-driven research, policy, and clinical practice that prioritizes the needs of historically marginalized communities.

Key priorities include standardising the reporting of race and ethnicity, incorporating intersectionality into research, and developing culturally sensitive diagnostic tools. Accurate prevalence estimates for EDs across diverse ethnic groups remain lacking. The acceptability, feasibility and effectiveness of current service and treatment models for minority ethnic individuals also require further study through RCTs and qualitative studies.

Although progress has been made, significant gaps persist in understanding the unique factors that influence the development, maintenance and treatment of EDs among minority ethnic populations and further work is needed to improve access to care and service provision. To advance equity, research and care must embrace inclusivity at every level, ultimately contributing to a healthcare system that serves all individuals equitably and effectively.

## Electronic supplementary material

Below is the link to the electronic supplementary material.

Additional File 1: Database Searches

Additional File 2: List of Excluded Articles

Additional File 3: Additional Tables

Additional File 4: PRISMA-ScR Checklist

## Data Availability

No datasets were generated or analysed during the current study.

## Footnotes

1 Three studies [94, 103, 107] assessed participants’ race or ethnicity on the basis of physical appearance (e.g., skin colour, facial features), though limited details of the exact procedures used were provided. The authors feel it is important to highlight this is highly problematic and such methodological choices may be reflective of the time period in which these studies were conducted (i.e., 1990–1996).

## Declarations

### Disclaimer

The views expressed are those of the authors and not necessarily those of the NIHR or the Department of Health and Social Care.

## Abbreviations

AN: Anorexia Nervosa
BED: Binge Eating Disorders
BN: Bulimia Nervosa
CBT: Cognitive Behavioural Therapy
DSM: Diagnostic and Statistical Manual of Mental Disorders
ED: Eating Disorder
EAT-26: Eating Attitude Test
GP: General Practitioner
ICD: International Classification of Diseases
IPT: Interpersonal Psychotherapy
NEDC: National Eating Disorders Collaboration
NICE: National Institute for Health and Care Excellence
NZMHS: New Zealand Mental Health Survey
OSFED: Other Specified Feeding or Eating Disorder
PRISMA-ScR: Preferred Reporting Items For Systematic Reviews And Meta-Analyses, Extension For Scoping Reviews
RCT: Randomised Control Trial
RANZCP: Royal Australian and New Zealand College of Psychiatrists
SES: Socioeconomic Status
UK: United Kingdom
US: United States
