# Supplementary material for: Eating disorders in minority ethnic populations in Australia, Canada, Aotearoa New Zealand and the UK: a scoping review
Source: J Eat Disord. 2025 Jan 14;13:8. doi: 10.1186/s40337-024-01173-y (PMC11734336; doi:10.1186/s40337-024-01173-y)
Supplement: Supplementary file 1 — Additional File 1: Database Searches [file 40337_2024_1173_MOESM1_ESM.docx]

**Additional File 1 – Database Searches**

**Medline**

Ovid MEDLINE(R) ALL <1946 to March 30, 2023>

1 "feeding and eating disorders"/ or anorexia nervosa/ or avoidant restrictive food intake disorder/ or binge-eating disorder/ or bulimia nervosa/ or diabulimia/ or "feeding and eating disorders of childhood"/ or food addiction/ or night eating syndrome/ or orthorexia nervosa/ or pica/ or relative energy deficiency in sport/ or female athlete triad syndrome/ or rumination syndrome/ 35804

2 eating disorder*.mp. 33463

3 anorexi*.mp. 42181

4 bulimi*.mp. 12324

5 ARFID.mp. 268

6 avoidant restrictive food intake disorder.mp. 432

7 binge eating.mp. 7407

8 pica.mp. 3495

9 rumination disorder.mp. 41

10 rumination syndrome.mp. 162

11 1 or 2 or 3 or 4 or 5 or 6 or 7 or 8 or 9 or 10 73164

12 population groups/ or african people/ or north african people/ or sub-saharan african people/ or central african people/ or east african people/ or southern african people/ or west african people/ or asian people/ or asian/ or central asian people/ or east asian people/ or north asian people/ or southeast asian people/ or west asian people/ or middle eastern people/ or south asian people/ or black people/ or "black or african american"/ or caribbean people/ or central american people/ or indians, central american/ or european people/ or eastern european people/ or "scandinavians and nordic people"/ or "middle eastern and north africans"/ or north american people/ or "american indian or alaska native"/ or indians, north american/ or alaskan natives/ or indigenous canadians/ or inuit/ or navajo people/ or pima people/ or population groups, us/ or ethnicity/ or "hispanic or latino"/ or mexican americans/ or racial groups/ or "native hawaiian or other pacific islander"/ or white/ or oceanians/ or australasian people/ or "australian aboriginal and torres strait islander peoples"/ or pacific island people/ or maori people/ or south american people/ or indians, south american/ or white people/ 326840

13 "emigrants and immigrants"/ or undocumented immigrants/ or refugees/ or "transients and migrants"/ 39912

14 "black or african american"/ or amish/ or arabs/ or "asian american native hawaiian and pacific islander"/ or asian/ or "native hawaiian or other pacific islander"/ or "hispanic or latino"/ or mexican americans/ or indigenous peoples/ or "american indian or alaska native"/ or indians, central american/ or indians, north american/ or alaskan natives/ or indigenous canadians/ or inuit/ or navajo people/ or pima people/ or indians, south american/ or "australian aboriginal and torres strait islander peoples"/ or maori people/ or roma/ 133629

15 asia, southern/ or afghanistan/ or bangladesh/ or bhutan/ or india/ or sikkim/ or maldives/ or nepal/ or pakistan/ or sri lanka/ 172233

16 ethnic minorit*.mp. 15411

17 racial minorit*.mp. 2337

18 multi-ethnic*.mp. 5370

19 "ethnic and racial minorities"/ or minority groups/ 17800

20 BAME.mp. 358

21 BIPOC.mp. 264

22 people of colo*r.mp. 1373

23 12 or 13 or 14 or 15 or 16 or 17 or 18 or 19 or 20 or 21 or 22 549059

24 11 and 23 1312

**EMBASE**

Embase Classic+Embase <1947 to 2023 Week 12>

1 eating disorder/ or anorexia nervosa/ or avoidant restrictive food intake disorder/ or binge eating disorder/ or bulimia/ or emotional eating/ or food addiction/ or food aversion/ or food neophobia/ or food refusal/ or muscle dysmorphia/ or orthorexia/ or pica/ or purging disorder/ or relative energy deficiency in sport/ 64869

2 (feeding and eating disorders).mp. [mp=title, abstract, heading word, drug trade name, original title, device manufacturer, drug manufacturer, device trade name, keyword heading word, floating subheading word, candidate term word] 5597

3 diabulimia.mp. 64

4 (feeding and eating disorders of childhood).mp. [mp=title, abstract, heading word, drug trade name, original title, device manufacturer, drug manufacturer, device trade name, keyword heading word, floating subheading word, candidate term word] 52

5 night eating syndrome.mp. 465

6 rumination syndrome/ or feeding disorder/ 6564

7 eating disorder*.mp. [mp=title, abstract, heading word, drug trade name, original title, device manufacturer, drug manufacturer, device trade name, keyword heading word, floating subheading word, candidate term word] 47823

8 anorexi*.mp. [mp=title, abstract, heading word, drug trade name, original title, device manufacturer, drug manufacturer, device trade name, keyword heading word, floating subheading word, candidate term word] 115739

9 bulimi*.mp. [mp=title, abstract, heading word, drug trade name, original title, device manufacturer, drug manufacturer, device trade name, keyword heading word, floating subheading word, candidate term word] 18145

10 ARFID.mp. [mp=title, abstract, heading word, drug trade name, original title, device manufacturer, drug manufacturer, device trade name, keyword heading word, floating subheading word, candidate term word] 438

11 avoidant restrictive food intake disorder.mp. [mp=title, abstract, heading word, drug trade name, original title, device manufacturer, drug manufacturer, device trade name, keyword heading word, floating subheading word, candidate term word] 698

12 binge eating.mp. [mp=title, abstract, heading word, drug trade name, original title, device manufacturer, drug manufacturer, device trade name, keyword heading word, floating subheading word, candidate term word] 12511

13 pica.mp. [mp=title, abstract, heading word, drug trade name, original title, device manufacturer, drug manufacturer, device trade name, keyword heading word, floating subheading word, candidate term word] 4755

14 rumination disorder.mp. [mp=title, abstract, heading word, drug trade name, original title, device manufacturer, drug manufacturer, device trade name, keyword heading word, floating subheading word, candidate term word] 57

15 rumination syndrome.mp. [mp=title, abstract, heading word, drug trade name, original title, device manufacturer, drug manufacturer, device trade name, keyword heading word, floating subheading word, candidate term word] 414

16 1 or 2 or 3 or 4 or 5 or 6 or 7 or 8 or 9 or 10 or 11 or 12 or 13 or 14 or 15 165879

17 population group/ or ancestry group/ or citizen group/ or ethnic group/ or minority group/ 107708

18 ancestry group/ or asian american/ or asian continental ancestry group/ or australoid/ or black person/ or british asian/ or caucasian/ or hispanic/ or indigenous people/ or migrant/ or mongoloid/ or multiracial person/ or oceanic ancestry group/ 279452

19 black person/ or african american/ or african brazilian/ or african caribbean/ 144776

20 caucasian/ or european american/ 172363

21 hispanic/ or mexican american/ 91634

22 indigenous people/ or alaska native/ or american indian/ or canadian aboriginal/ or first nation/ or indigenous australian/ or taiwanese aborigine/ 35946

23 migrant/ or emigrant/ or forced migrant/ or immigrant/ or migrant worker/ 34289

24 multiracial person/ 711

25 oceanic ancestry group/ or pacific islander/ or torres strait islander/ 10630

26 citizen group/ or african/ or asian/ or "caribbean (person)"/ or central american/ or european/ or north american/ or oceanian/ or south american/ 255326

27 african/ or central african/ or east african/ or north african/ or southern african/ or west african/ 11807

28 central african/ or angolan/ or cameroonian/ or "chadian (citizen)"/ or "citizen of the central african republic"/ or "congolese (brazzaville)"/ or "congolese (kinshasa)"/ or gabonese/ 855

29 east african/ or eritrean/ or ethiopian/ or kenyan/ or "malagasy (citizen)"/ or malawian/ or mauritian/ or mozambican/ or rwandan/ or "somali (citizen)"/ or tanzanian/ or ugandan/ or zambian/ or zimbabwean/ 6458

30 north african/ or algerian/ or egyptian/ or libyan/ or moroccan/ or sudanese/ or tunisian/ 11694

31 southern african/ or namibian/ or south african/ or "swazi (citizen)"/ 4278

32 west african/ or beninese/ or burkinabe/ or gambian/ or ghanaian/ or guinean/ or "ivorian (citizen)"/ or liberian/ or malian/ or mauritanian/ or nigerian/ or nigerien/ or senegalese/ or sierra leonean/ or togolese/ 5473

33 asian/ or central asian/ or east asian/ or south asian/ or southeast asian/ or west asian/ 106199

34 central asian/ or kazakhstani/ or "kyrgyz (citizen)"/ or "tajik (citizen)"/ or "turkmen (citizen)"/ or "uzbek (citizen)"/ 405

35 east asian/ or chinese/ or "japanese (citizen)"/ or "mongolian (citizen)"/ or north korean/ or south korean/ or taiwanese/ 93841

36 south asian/ or afghan/ or bangladeshi/ or bhutanese/ or indian/ or nepalese/ or pakistani/ or sri lankan/ 51986

37 southeast asian/ or burmese/ or cambodian/ or "filipino (citizen)"/ or indonesian/ or laotian/ or malaysian/ or singaporean/ or "thai (citizen)"/ or vietnamese/ 11375

38 west asian/ or "armenian (citizen)"/ or azerbaijani/ or bahraini/ or cypriot/ or emirati/ or "georgian (citizen)"/ or "iranian (citizen)"/ or iraqi/ or israeli/ or jordanian/ or kuwaiti/ or lebanese/ or omani/ or palestinian/ or qatari/ or saudi/ or syrian/ or turkish citizen/ or yemeni/ 16521

39 caribbean/ or antillean/ 5271

40 central american/ or belizean/ or costa rican/ or guatemalan/ or honduran/ or mexican/ or nicaraguan/ or panamanian/ or salvadoran/ 6159

41 european/ or central european/ or eastern european/ or eu citizen/ or northern european/ or southern european/ or western european/ 146844

42 central european/ or austrian/ or "czech (citizen)"/ or "german (citizen)"/ or "hungarian (citizen)"/ or polish citizen/ or "slovak (citizen)"/ or "slovenian (citizen)"/ or swiss/ 18342

43 eastern european/ or "armenian (citizen)"/ or azerbaijani/ or "belarusian (citizen)"/ or "bulgarian (citizen)"/ or "czech (citizen)"/ or "georgian (citizen)"/ or "hungarian (citizen)"/ or polish citizen/ or "romanian (citizen)"/ or "russian (citizen)"/ or "slovak (citizen)"/ or "ukrainian (citizen)"/ 9113

44 eu citizen/ or austrian/ or belgian/ or "bulgarian (citizen)"/ or "croatian (citizen)"/ or cypriot/ or "czech (citizen)"/ or danish citizen/ or dutchman/ or "estonian (citizen)"/ or "finn (citizen)"/ or frenchman/ or "german (citizen)"/ or "greek (citizen)"/ or "hungarian (citizen)"/ or "irish (citizen)"/ or "italian (citizen)"/ or "latvian (citizen)"/ or "lithuanian (citizen)"/ or "maltese (citizen)"/ or polish citizen/ or "portuguese (citizen)"/ or "romanian (citizen)"/ or "slovak (citizen)"/ or "slovenian (citizen)"/ or spaniard/ or swedish citizen/ 52321

45 northern european/ or british citizen/ or danish citizen/ or "estonian (citizen)"/ or faroese/ or "finn (citizen)"/ or icelander/ or "irish (citizen)"/ or "latvian (citizen)"/ or "lithuanian (citizen)"/ or "norwegian (citizen)"/ or swedish citizen/ 14870

46 british citizen/ or briton/ or northern irish/ 1731

47 briton/ or englishman/ or scotsman/ or welshman/ 1166

48 southern european/ or "albanian (citizen)"/ or "bosnian (citizen)"/ or "croatian (citizen)"/ or cypriot/ or "greek (citizen)"/ or "italian (citizen)"/ or kosovar/ or "macedonian (citizen)"/ or "maltese (citizen)"/ or "portuguese (citizen)"/ or "serbian (citizen)"/ or "slovenian (citizen)"/ or spaniard/ or yugoslav/ 18026

49 western european/ or austrian/ or belgian/ or dutchman/ or frenchman/ or "german (citizen)"/ or swiss/ 22080

50 north american/ or american/ or canadian/ 173924

51 oceanian/ or australian/ or melanesian/ or micronesian/ or new zealander/ or polynesian/ 16781

52 melanesian/ or fijian/ or new caledonian/ or papua new guinean/ 276

53 micronesian/ or marshallese/ or nauruan/ or palauan/ 94

54 polynesian/ or american samoan/ or cook islander/ or french polynesian/ or "hawaiian (citizen)"/ or tongan/ or western samoan/ 406

55 south american/ or argentinian/ or bolivian/ or brazilian/ or chilean/ or colombian/ or ecuadorean/ or guianese/ or guyanese/ or paraguayan/ or peruvian/ or surinamese/ or uruguayan/ or venezuelan/ 17847

56 ethnic group/ or afro-asiatic people/ or altaic people/ or amerind people/ or amish/ or australian aborigine/ or austroasiatic people/ or austronesian people/ or "basque (people)"/ or caucasian speaking people/ or dravidian people/ or eskimo-aleut people/ or "hadza (people)"/ or hmong-mien people/ or indo-european people/ or khoisan/ or na-dene people/ or negrito/ or niger-congo people/ or nilo-saharan people/ or paleosiberian people/ or papuan people/ or pygmy/ or sino-tibetan people/ or tai-kadai people/ or uralic people/ 85845

57 ethnic group/ or exp afro-asiatic people/ or exp altaic people/ or exp amerind people/ or exp amish/ or exp australian aborigine/ or exp austroasiatic people/ or exp austronesian people/ or exp "basque (people)"/ or exp caucasian speaking people/ or exp dravidian people/ or exp eskimo-aleut people/ or exp "hadza (people)"/ or exp hmong-mien people/ or exp indo-european people/ or exp khoisan/ or exp na-dene people/ or exp negrito/ or exp niger-congo people/ or exp nilo-saharan people/ or exp paleosiberian people/ or exp papuan people/ or exp pygmy/ or exp sino-tibetan people/ or exp tai-kadai people/ or exp uralic people/ 190016

58 ethnic minorit*.mp. [mp=title, abstract, heading word, drug trade name, original title, device manufacturer, drug manufacturer, device trade name, keyword heading word, floating subheading word, candidate term word] 20056

59 racial minorit*.mp. [mp=title, abstract, heading word, drug trade name, original title, device manufacturer, drug manufacturer, device trade name, keyword heading word, floating subheading word, candidate term word] 2662

60 multi-ethnic*.mp. [mp=title, abstract, heading word, drug trade name, original title, device manufacturer, drug manufacturer, device trade name, keyword heading word, floating subheading word, candidate term word] 9364

61 BAME.mp. [mp=title, abstract, heading word, drug trade name, original title, device manufacturer, drug manufacturer, device trade name, keyword heading word, floating subheading word, candidate term word] 595

62 BIPOC.mp. [mp=title, abstract, heading word, drug trade name, original title, device manufacturer, drug manufacturer, device trade name, keyword heading word, floating subheading word, candidate term word] 332

63 17 or 18 or 19 or 20 or 21 or 22 or 23 or 24 or 25 or 26 or 27 or 28 or 29 or 30 or 31 or 32 or 33 or 34 or 35 or 36 or 37 or 38 or 39 or 40 or 41 or 42 or 43 or 44 or 45 or 46 or 47 or 48 or 49 or 50 or 51 or 52 or 53 or 54 or 55 or 56 or 57 or 58 or 59 or 60 or 61 or 62 1199105

64 16 and 63 6820

**PsycInfo**

APA PsycInfo <1806 to March Week 3 2023>

1 eating disorders/ or anorexia nervosa/ or "avoidant/restrictive food intake disorder"/ or binge eating disorder/ or bulimia/ or feeding disorders/ or hyperphagia/ or kleine levin syndrome/ or orthorexia/ or pica/ or "purging (eating disorders)"/ or "rumination (eating)"/ or binge eating/ or food addiction/ 36303

2 (feeding and eating disorders).mp. [mp=title, abstract, heading word, table of contents, key concepts, original title, tests & measures, mesh word] 9219

3 diabulimia.mp. 12

4 (feeding and eating disorders of childhood).mp. [mp=title, abstract, heading word, table of contents, key concepts, original title, tests & measures, mesh word] 229

5 night eating syndrome.mp. 267

6 relative energy deficiency in sport.mp. 6

7 eating disorder*.mp. 35849

8 anorexi*.mp. 19945

9 bulimi*.mp. 14423

10 ARFID.mp. 243

11 avoidant restrictive food intake disorder.mp. 334

12 binge eating.mp. 7879

13 pica.mp. 736

14 rumination disorder.mp. 80

15 rumination syndrome.mp. 27

16 1 or 2 or 3 or 4 or 5 or 6 or 7 or 8 or 9 or 10 or 11 or 12 or 13 or 14 or 15 50512

17 "racial and ethnic groups"/ or african cultural groups/ or asians/ or blacks/ or caribbean cultural groups/ or european cultural groups/ or indigenous populations/ or "latinos/latinas"/ or "middle eastern and north african cultural groups"/ or multiracial/ or "people of color"/ or romanies/ or whites/ or cross cultural differences/ or cultural sensitivity/ or ethnic diversity/ or ethnic values/ or multiculturalism/ or "race (anthropological)"/ or "racial and ethnic attitudes"/ or "racial and ethnic differences"/ or racial disparities/ or racial identity/ or systemic racism/ 206179

18 asians/ or chinese cultural groups/ or japanese cultural groups/ or korean cultural groups/ or south asian cultural groups/ or southeast asian cultural groups/ or vietnamese cultural groups/ 28904

19 japanese cultural groups/ or japanese americans/ 1847

20 southeast asian cultural groups/ or vietnamese cultural groups/ 2159

21 indigenous populations/ or alaska natives/ or american indians/ or inuit/ or pacific islanders/ 15920

22 pacific islanders/ or hawaii natives/ 1152

23 "latinos/latinas"/ or mexican americans/ 33166

24 "middle eastern and north african cultural groups"/ or arabs/ 3975

25 whites/ or anglos/ 21972

26 immigration/ or undocumented immigration/ or expatriates/ or human migration/ or migrant workers/ or refugees/ 41412

27 ethnic minorit*.mp. [mp=title, abstract, heading word, table of contents, key concepts, original title, tests & measures, mesh word] 14105

28 racial minorit*.mp. [mp=title, abstract, heading word, table of contents, key concepts, original title, tests & measures, mesh word] 2386

29 multi-ethnic*.mp. [mp=title, abstract, heading word, table of contents, key concepts, original title, tests & measures, mesh word] 1570

30 BAME.mp. [mp=title, abstract, heading word, table of contents, key concepts, original title, tests & measures, mesh word] 121

31 BIPOC.mp. [mp=title, abstract, heading word, table of contents, key concepts, original title, tests & measures, mesh word] 222

32 people of colo*r.mp. [mp=title, abstract, heading word, table of contents, key concepts, original title, tests & measures, mesh word] 2859

33 17 or 18 or 19 or 20 or 21 or 22 or 23 or 24 or 25 or 26 or 27 or 28 or 29 or 30 or 31 or 32 269191

34 16 and 33 1587

**CINAHL**

Top of Form

| **#** | **Query** | **Limiters/Expanders** | **Last Run Via** | **Results** |
| --- | --- | --- | --- | --- |
| S6 | S1 AND S5 | Expanders - Apply equivalent subjects Search modes - Find all my search terms | Interface - EBSCOhost Research Databases Search Screen - Advanced Search Database - CINAHL | 1,018 |
| S5 | S2 OR S3 OR S4 | Expanders - Apply equivalent subjects Search modes - Find all my search terms | Interface - EBSCOhost Research Databases Search Screen - Advanced Search Database - CINAHL | 186,914 |
| S4 | ethnic minority or racial minority or BAME or BIPOC | Expanders - Apply equivalent subjects Search modes - Boolean/Phrase | Interface - EBSCOhost Research Databases Search Screen - Advanced Search Database - CINAHL | 10,473 |
| S3 | (MH "Immigrants") OR (MH "Undocumented Immigrants") OR (MH "Transients and Migrants") | Expanders - Apply equivalent subjects Search modes - Boolean/Phrase | Interface - EBSCOhost Research Databases Search Screen - Advanced Search Database - CINAHL | 23,520 |
| S2 | (MH "Ethnic Groups+") OR (MH "Amish") OR (MH "Arabs") OR (MH "Asians") OR (MH "Cambodians") OR (MH "Chinese") OR (MH "Filipinos") OR (MH "Hmong") OR (MH "Japanese") OR (MH "Koreans") OR (MH "Laotians") OR (MH "Thais") OR (MH "Vietnamese") OR (MH "Black Persons") OR (MH "African Americans") OR (MH "Roma") OR (MH "Hispanic Americans") OR (MH "Mexican Americans") OR (MH "Indigenous Peoples") OR (MH "Aboriginal Canadians") OR (MH "First Nations of Canada") OR (MH "Arctic Peoples") OR (MH "Inuit") OR (MH "First Nations of Australia") OR (MH "Aboriginal Australians") OR (MH "Torres Strait Islanders") OR (MH "Maori") OR (MH "Native Americans") OR (MH "Alaska Natives") OR (MH "Jews") OR (MH "Kurds") OR (MH "White Persons") | Expanders - Apply equivalent subjects Search modes - Boolean/Phrase | Interface - EBSCOhost Research Databases Search Screen - Advanced Search Database - CINAHL | 166,196 |
| S1 | (MH "Eating Disorders+") OR (MH "Eating Disorders Management (Iowa NIC)") OR (MH "Binge Eating Disorder") OR (MH "Feeding and Eating Disorders of Childhood+") OR (MH "Bulimia Nervosa") OR (MH "Avoidant Restrictive Food Intake Disorder") OR (MH "Bulimia") OR "eating disorders or anorexia or bulimia or disordered eating or binge eating disorder | Expanders - Apply equivalent subjects Search modes - Boolean/Phrase | Interface - EBSCOhost Research Databases Search Screen - Advanced Search Database - CINAHL | 29,448 |

Bottom of Form

**Web of Science**

(TS=("eating disorder" OR anorexia OR bulimia OR "binge eating" OR pica OR "rumination disorder" OR "disordered eating" OR "eating disorders" OR ARFID OR "avoidant restrictive food intake disorder")) AND TS=(ethnicity OR "ethnic group" OR ethnic OR african OR asian OR black OR caribbean OR "central american" OR european OR "middle eastern" OR "north american" OR latino OR oceanian OR australian OR white OR caucasian OR immigrant OR migrant OR refugee OR "racial group" OR "racial minority" OR "ethnic minority" OR multi-ethnic OR BAME OR BIPOC )

**4993 results**
