# Supplementary material for: Eating disorders in minority ethnic populations in Australia, Canada, Aotearoa New Zealand and the UK: a scoping review
Source: J Eat Disord. 2025 Jan 14;13:8. doi: 10.1186/s40337-024-01173-y (PMC11734336; doi:10.1186/s40337-024-01173-y)
Supplement: Supplementary file 3 — Additional File 3: Additional Tables [file 40337_2024_1173_MOESM3_ESM.docx]

## **Additional File 3 – Additional Tables**

## **Table S1** Study and sample characteristics

| **First Author & Publication Year** | **Country** | **Study Design** | **Sample Type** | **Eating Disorder/ Disordered Eating** | **Sample Size (N)** | **Sex** | **Age (Years)** | **Ethnicity**^a^ | **Religion** | **Socioeconomic Status (SES)** | **Co-morbidity** |
| --- | --- | --- | --- | --- | --- | --- | --- | --- | --- | --- | --- |
| Lake et al. (2000)  (51) | Australia | Cross-sectional survey | Non-clinical | Disordered eating (EAT-26) | 140 | 140F  (100% female) | R: 17-43 Australian born sample: M: 20.13, SD: 4.32 Hong Kong born sample: M: 23.24, SD: 5.30 | *Participant place of birth:*  Australia 70% Hong Kong 30% | NR | NR | NR |
| Rieger et al. (2000)  (52) | Australia | Retrospective case review | Clinical | AN (n = 8) BN (n = 6) | 14 | NR | M: 21.6 | Asian 100% (Hong Kong n = 6, Japan n = 3, Singapore n = 2, Malaysia n = 2, Indonesia n = 1) | NR | *Educational attainment:* mean level of education 12.3 years | NR |
| Ball & Kennardy (2002)  (53) | Australia | Cross-sectional survey | Non-clinical | Disordered eating (adapted EDE-S) | 14,779 | 14,779F (100% female) | R: 18-23 | *Participant place of birth:* Asia 3% Australia 91% Europe 1% Other English speaking country 4% Other non-English speaking country 1% | NR | NR | NR |
| Humphry & Ricciardelli (2004)  (54) | Australia | Cross-sectional survey | Non-clinical | Disordered eating (EAT-26) | 81 | 81F  (100% female) | M: 28.55 | *Participant and parent country of birth:* China 100% | NR | NR | NR |
| Jennings et al. (2005)  (55) | Australia | Cross-sectional survey | Non-clinical | Disordered eating (EAT-26, EDI-2, EDI-SC) | 42 | 42F  (100% female) | R: 14-17 Asian sample:  M: 15.4, SD: 0.9  Caucasian sample:  M; 14.9, SD: 0.6 | Asian 40% Caucasian 60% | Asian sample: Christian 82% Caucasian sample: Christian 52%, atheist 40% | NR | NR |
| Wang et al. (2005)  (56) | Australia | Cross-sectional survey | Non-clinical | Disordered eating (EAT-26) | 768 | 529F/239M (69% female) | R: 10-18 M: 13.8 SD: 2.0 | Caucasian 75% Chinese/Vietnamese 18% Italian/Greek 7% | NR | *Parent's occupation:* High SES 82% Middle SES 9% Low SES 9% | NR |
| Jennings et al. (2006a)  (57) | Australia | Cross-sectional survey | Non-clinical | Disordered eating (EAT-26, EDI-2, EDI-SC) | 341 | 341F  (100% female) | R: 18-24 Asian Australian sample: M: 19.3, SD: 1.5 Caucasian Australian sample:  M: 19.2, SD: 1.3 Thai sample:  M: 19.5, SD: 1.0 | *Self-report:* Asian Australian 38% Caucasian Australian 32% Thai 30% | Asian Australian sample:  Christian 45% Caucasian Australian sample: Christian 56% Thai sample: Buddhist 99% | *ASCO (father's occupation):* Asian Australian sample: professional 36% Caucasian Australian sample: professional 47% Thai sample: professional 18% | NR |
| Jennings et al. (2006b)  (58) | Australia | Cross-sectional survey | Non-clinical | Disordered eating (EAT-26, EDI-2, EDI-SC) | 240 | 240F  (100% female) | R: 18-24 Asian sample:  M: 19.3, SD: 1.5 Caucasian sample:  M: 19.2, SD: 1.3 | Asian 54% (Chinese n = 42, Malaysian n = 26, other Asian n = 62) Caucasian 46% | NR | NR | NR |
| Soh et al. (2007)  (59) | Australia | Cross-sectional survey | Mixed: clinical and non-clinical | *DSM-IV diagnostic criteria:* AN 71%, BN 24%, EDNOS 5% Disordered eating (EDE-Q) | 154 (55 ED patients; 99 controls) | 154F  (100% female) | R: 14-38 M: 21.3 | *Self-report:* North European Australian 43% East Asian Australian (Chinese, Vietnamese, Korean) 16% Singaporean Chinese 33% North European expatriates in Singapore 8% | NR | *1-7 prestige scale (parent occupation):* mean rating 3.82 | NR |
| Hay & Carriage (2012)  (60) | Australia | Secondary data analysis | Non-clinical | ED symptoms (adapted EDE) | 6081 | 3108F/2973M (51% female) | R: ≥ 15  Indigenous sample:  M: 39.2, SD: 16.7 (2005); M: 37.4, SD: 18.3 (2008) non-Indigenous sample: M: 45.3, SD: 18.8 (2005); M: 46.0, SD: 8.9 (2008) | *Self-report:* First Australian 3% other Australian (non-Indigenous) 97% | NR | *Median household income & educational attainment:* Indigenous sample:  > $40-50,000 (2005), > $80-100,000 (2008); trade qualification/apprenticeship (2005), certificate/diploma (2008) non-Indigenous sample:  > $50-60,000 (2005),  > $60-80,000 (2008); certificate/diploma (2005 & 2008) | NR |
| Mulders-Jones et al. (2017)  (61) | Australia | Secondary data analysis | Non-clinical | ED symptoms (adapted EDE) | 6041 | 3081F/2960M (51% female) | R: ≥ 15  M: 45.6 SD: 18.9 | *Self-report:* Aboriginal 2% Torres Strait Islander (n = 4) Aboriginal and Torres Strait Islander (n = 1) non-Indigenous (74%) Prefer not to answer (26%) | NR | *Household income, educational attainment, employment status, geographic location:* household income >$60,000 50%; left school/still studying 40%; full time employment 38%; living in high accessibility areas 76% | NR |
| Cheah et al. (2020)  (62) | Australia | Secondary data analysis | Non-clinical | ED symptoms (adapted EDE) DSM-5 diagnostic criteria | 6052 | 3076F/2976M (51% female) | R: ≥ 15  Australian born sample: M: 45.96 First-generation migrant sample: M: 48.93 | *Participant place of birth:* Australian born 72% Born outside Australia 28% (Africa n = 91, America n = 47, Asia n = 605, Germany n = 45, Greece n = 32, Holland n = 39, Italy n = 67, New Zealand or Oceania n = 80, other European n = 134, UK & Ireland n = 579) | NR | *Household income & educational attainment:*  Australia born sample:  > $60,000 43%; bachelor or higher degree 16% First-generation migrant sample:  > $60,000 35%; bachelor or higher degree 28% | NR |
| Burt et al. (2020a)  (63) | Australia | Secondary data analysis | Non-clinical | ED symptoms (adapted EDE) DSM-5 diagnostic criteria | 6052 | 3076F/2976M (51% female) | R: ≥ 15  First Australian sample: M: 36.49, SD: 3.59 (with ED); M: 37.54, SD: 3.26 (no ED) other Australian sample: M: 39.98, SD: 2.03 (with ED); M: 48.48, SD: 2.42 (no ED) | *Self-report:* First Australian (Aboriginal and/or Torres Strait Islander) 2% other Australian (non-Indigenous) 98% | NR | NR | NR |
| Burt et al. (2020b)  (64) | Australia | Secondary data analysis | Non-clinical | Disordered eating (EDE-Q) NES symptoms (NEQ) DSM-5 diagnostic criteria | 5068 | 2065F/3003M (41% female) | First Australian sample: M: 15.0, SD: 1.30 (with ED); M: 14.4, SD: 1.5 (no ED) other Australian sample: M: 15.2, SD: 1.4 (with ED); M: 14.7, SD: 1.4 (no ED) | *Self-report:* First Australian (Aboriginal and/or Torres Strait Islander) 8% other Australian (non-Indigenous) 91% Declined to answer 1.6% | NR | *Mean SEFIA score:* Indigenous sample: 975.0, SD: 43.4 (with ED); 965.1, SD: 42.1 (no ED) other Australian sample: 987.4, SD: 40.9 (with ED); 988.3, SD: 42.3 (no ED) | NR |
| Geller (1996)*  (65) | Canada | Cross-sectional survey | Non-clinical | Bulimic symptoms (BULIT-R) | 79 | 79F  (100% female) | R: 17-50 M: 23.6 SD: 6.5 | *Self-report:* Caucasian 47% Native/Aboriginal 35% (Metis n = 9, Inuit n = 1, non-status n = 5, status including members of the Cayuga, Cree, First Nations, Iroquois, Ojibway and Oneida n = 13) Born outside North America 18% (Arab n = 1, Chinese n = 8, European n = 5) | NR | *Estimated family income & Blishen 1987 index:*  mean income $16,756.9, SD: $4310.8; mean SES index score 50.4, SD: 12.1 | NR |
| Tessier (2001)*  (66) | Canada | Cross-sectional survey | Non-clinical | Disordered eating (EDI-2) | 454 | 244F/210M (54% female) | R: 11-19 M: 14.68 | *Self-report:* Aboriginal 11% African 2% Asian 3% Canadian 31% European 53% | NR | NR | NR |
| Birmingham and Sidhu (2007)  (67) | Canada | Case study | Clinical | AN | 1 | 1F | 38 | Southeast Asian | NR | NR | NR |
| Boisvert & Harrell (2009)  (68) | Canada | Population survey | Non-clinical | Disordered eating (EDI items) | 601 | 601F (100% female) | R: ≥18 | *Self-report:* Aboriginal 2.5% Black 0.5% Asian 4.6% Hispanic 2.5% White 89% | NR | NR | NR |
| Boisvert & Harrell (2012)  (69) | Canada | Population survey | Non-clinical | Disordered eating (EDI items) | 603 | 603M  (0% female) | R: ≥18  M: 42.33  SD: 15.44 | *Self-report:* Aboriginal 2.4% Asian 6.0% Hispanic 2.5% White 86% | NR | NR | NR |
| Buttu (2012)*  (70) | Canada | Cross-sectional survey | Non-clinical | Bulimic symptoms (BULIT-R) Disordered eating (EAT-26) | 196 | 196F (100% female) | R: 18-35 M: 23.77 SD: 4.87 | *Self-report:* Arab-Canadian 100% | atheist 4% Christian 20% Muslim 70% other religion 5% not specified 2% | *Educational attainment & employment status:* some university education 31.6%; full-time employment 30.6% | NR |
| Boisvert & Harrell (2013)  (71) | Canada | Population survey | Non-clinical | Disordered eating (EDI items) | 591 | 591F (100% female) | R: ≥18 years | *Self-report:* Aboriginal 2.6% Asian 4.7% Hispanic 2.5% White 90% | NR | NR | NR |
| Boisvert & Harrell (2014)  (72) | Canada | Population survey | Non-clinical | Disordered eating (EDI items) | 1204 | 601F/603M (50% female) | R: ≥18  M: 43.1 SD: 15.95 | *Self-report:* White (mainly European heritage) 88% non-White (Asian, Black, Hispanic, Native American-Indian) 12% | NR | NR | NR |
| Mustafa et al. (2016)  (73) | Canada | Qualitative study | Clinical | AN (n = 5) BN (n = 1) BED (n = 2) | 8 | 8F (100% female) | R: 21-29 | *Self-report:* South Asian 100% (Indian n = 7, Pakistani n = 1) | Hindu 37.5% Muslim 50% Sikh 12.5% | *Educational attainment:* post-secondary qualification 100% | NR |
| Cheung & Wilder-Smith (1995)  (74) | New Zealand | Case study | Clinical | *DSM-III-R and ICD-10 diagnostic criteria:* AN | 1 | 1M | 22 | P*articipant place of birth:* Chinese | NR | NR | Schizophrenia (DSM-III-R) |
| Chan & Owens (2006)  (75) | New Zealand | Cross-sectional survey | Non-clinical | Disordered eating (EDI) | 301 | 179F/122M (60% female) | M: 22.37 | *Parental ethnicity:* Chinese 100% | NR | NR | NR |
| Oakley-Browne et al. (2006)  (76) | New Zealand | Population survey | Non-clinical | DSM-IV diagnostic criteria | 12,992 | 7358F/5454M (57% female) | R: ≥ 16 | *Self-report (census categories):*  Māori 20% Pacific 17% other ethnicity (predominantly European descent) 63% | NR | NR | DSM-IV psychiatric conditions: anxiety disorders, mood disorders & substance abuse Lifetime prevalence of two mental disorders 9.9% (95% CI 9.2-10.6); three or more mental disorders 9.7% (95% CI 9.0-10.4) |
| Baxter et al. (2006)  (77) | New Zealand | Population survey | Non-clinical | DSM-IV diagnostic criteria | 2595 | 1547F/1048M (60% female) | R: ≥ 16 | *Self-report (census categories):* Māori 100% (37.1% identified as Māori in addition to another ethnic group) | NR | *Educational attainment, household income & NZDep 2001:* no qualifications 31.8%, household income < half the median 31.8%, decile 9 and 10 43.7% | DSM-IV disorders: anxiety disorders, mood disorders & substance abuse Lifetime prevalence of two mental disorders 13.8% (95% CI 12.0-15.8); three or more disorders 17.1% (95% CI 15.2-19.2) |
| Foliaki et al. (2006)  (78) | New Zealand | Population survey | Non-clinical | DSM-IV diagnostic criteria | 2374 | 1234F/1140M (52% female) | R: ≥ 16 | *Self-report (census categories):* Pacific 100% (Cook Island 20.7%, Samoan 49.2%, Tongan 16.5%, other 17.8%) | NR | *Educational attainment, household income & NZDep2001:* no qualifications 24.6%, household income < half the median 28.7%, decile 9 and 10 59% | DSM-IV disorders: anxiety disorders, mood disorders & substance abuse Lifetime prevalence of two mental disorders 12.4% (95% CI 10.3-14.9); three disorders 10.7% (95% CI 8.7-13.0) |
| Ngamanu (2006)*  (79) | New Zealand | Cross-sectional survey | Non-clinical | Disordered eating (EAT-26) | 100 | 100F (100% female) | R: 18-50+ 55% > 35, 9% > 50 | *Self-report:* Caucasian/Pakeha 41% Māori 28% Māori and Pakeha 21% | NR | NR | NR |
| Jenkins (2007)*  (80) | New Zealand | Cross-sectional survey | Non-clinical | Disordered eating (EAT-26, EAT-40, SEED) | 116 | 116F (100% female) | R: 18-47 Chinese sample:  M: 24.9, SD: 3.51 other ethnicity sample: M: 24.12, SD: 8.66 | *Self-report:* Chinese 39% (Chinese n = 39, Taiwan n = 6) other ethnicity 61% (Māori n = 9, New Zealand Europeans n = 57, Pacific Islander n = 1, other ethnicity n = 4) | NR | NR | NR |
| Chan et al. (2010)  (81) | New Zealand | Cross-sectional survey | Non-clinical | Disordered eating (EDI) | 123 | 72F/51M (60% female) | R: 12-58 M: 24.48 SD: 11.55 | *Parental ethnicity:* Korean 100% | NR | *Educational attainment:* primary school 2.4%, high school 28.5%, university entrance/bursary 44.7%, tertiary 13.8%, postgraduate 8.9% | NR |
| Davey (2012)*  (82) | New Zealand | Quasi-experimental study | Clinical | AN (n = 103), BN (n = 55), EDNOS (n = 89), no diagnosis (n = 5) Disordered eating (EDE-Q) | 252 | 244F/8M (97% female) | R: 11-62 | Asian 4.8% European 18.3% New Zealand European 70% Māori 4.8% Middle Eastern 0.8% Pacific 0.8% South American 0.4% | NR | NR | DSM-IV-TR disorders: mood disorder (n = 109); anxiety disorder (n = 69); substance abuse/ dependency (n = 35); personality disorder (n = 16); other (ADHD, trichotillomania, BDD, psychosis) (n = 10) |
| Lacey et al. (2020)  (83) | New Zealand | Secondary data analysis | Clinical | *DSM-IV or ICD-10 diagnostic criteria:* non-Māori sample: AN 41.5%; AN & BN 5.7%; BN 18.1%; OSFED 0.7%; EDNOS 33.9% Māori sample: AN 27.8%; AN & BN 4.2%; BN 24.3%, OSFED 0.4%; EDNOS 43.3% | 3835 | 3356F/479M (94% female) | R: 10-65 | *Self-report:* non-Māori 93% (Asian n = 195, European/other n = 3337, Pacific n = 41) Māori 7% | NR | *Deprivation quintiles:* non-Māori sample: quintiles 1 and 2 48.5% Māori sample:  quintiles 4 and 5 47.7% | other psychiatric disorders (Māori/non- Māori):  schizophrenia 6.1%/3,1%; bipolar 4.6%/4.2%; depression 47.9%/38%; anxiety 43.7%/37%; alcohol/substance abuse 24%/15.1%; personality disorder 27.5%/11.2%; intellectual disability 6.1%/4.1%; other disorder 7.2%/4.3%; no other diagnosis 32.7%/41.8% |
| Clark et al. (2023)  (84) | New Zealand | Qualitative Study | Mixed: clinical and relatives | *DSM-IV and ICD-10 diagnostic criteria:* AN (n = 8), BN (n = 4), BED (n = 1) | 15 (13 ED patients; 2 relatives) | 14F/1M (93% female) | R: 16-65 | *Self-report:* Māori 100% | NR | NR | NR |
| Thomas & Szmukler (1985)  (85) | UK | Case series | Clinical | *Russell (1970, 1979) diagnostic criteria:* AN (n = 1) BN (n = 1) AN & BN (n = 1) | 3 | 3F (100% female) | R: 17-20 | *Parental/family ethnicity:* Afro-Caribbean 100% (Barbadian n = 1, Jamaican n = 2) | NR | Working class 100% | NR |
| Nasser (1986)  (86) | UK | Two-phase prevalence study | Non-clinical | Disordered eating (EAT-40) Russell (1979) diagnostic criteria | 110 UK sample: 50 Cairo sample: 60 | 110F (100% female) | Cairo sample: M: 23.4, SD: 2.5 London sample: M: 21.8, SD: 2.8 | Arab 100% | NR | *OPCS (father's occupation):* London sample: Class I and II Cairo sample: matched for social class | NR |
| Mumford & Whitehouse (1988)  (87) | UK | Two-phase prevalence study | Non-clinical | Disordered eating (EAT-26) DSM-III-R diagnostic criteria | 559 | 559F (100% female) | Whole sample: R: 14-16  Asian sample:  M: 15.1, SD: 1.6 Caucasian sample:  M: 14.9, SD: 1.0 | Caucasian 64% South Asian 36% | NR | NR | NR |
| Holden & Robinson (1988)  (88) | UK | Retrospective case review | Clinical | *Russell (1970, 1983) diagnostic criteria:* Black sample: AN (n = 2), BN (n = 11) White sample: AN (n = 2), BN (n = 11) | 26 | 26F (100% female) | R: 16-28 | Black 50% (n = 1 African; n = 1 African & German; n = 8 West Indian; n = 3 West Indian & White British) White 50% | NR | *OPCS (patient occupation):* Black sample: Class II 15%; Class III 69%; Class IV 8%; Class V 8% White sample: Class II 46%; Class III 38%; Class IV 8%; Class V 8% | other psychiatric disorder (Black/ White):  depression (n = 11/8), substance abuse (n = 4/1); parasuicide (n = 2/ 1) |
| Lacey & Dolan (1988)  (89) | UK | Case series | Clinical | *DSM-III-R diagnostic criteria:* BN (n = 5) | 5 | 5F (100% female) | R: 18-26 | *Parental birthplace:* Jamaican (n = 1) Mixed racial background (n = 2 Afro-Caribbean & White British; n = 1 African & Indian) Pakistani (n = 1) | NR | NR | depression (n = 1); alcohol abuse (n = 1) |
| Fahy et al. (1988)  (90) | UK | Case study | Clinical | AAN  Disordered eating (EAT-26) | 1 | 1F | 22 | *Participant birthplace:* Ethiopian | NR | NR | anxiety (symptoms); depression (symptoms) |
| Ford & Dolan (1989)  (91) | UK | Case study | Clinical | BN | 1 | 1F | 32 | Asian | Sikh | NR | asthma, spontaneous abortion, depression, self-harm, suicidal attempts, substance abuse |
| Bhadrinath (1990)  (92) | UK | Case series | Clinical | DSM-III-R diagnostic criteria: AN (n = 3) | 3 | 2F/1M (67% female) | R: 15-16 | *Participant birthplace:*  Pakistani (n = 2) Kenyan Asian (n = 1) | Muslim 67% Sikh 33% | Working class 66% Middle class 33% | DSM-III-R depression (n = 1) |
| Dolan et al. (1990)  (93) | UK | Cross-sectional survey | Non-clinical | Disordered eating (EAT-26) | 479 | 479F (100% female) | M: 28.2 | *Self-report:* Afro-Caribbean 15% Asian (including East African Asian, Indian, Pakistani, Sri Lankan) 9% Caucasian 76% | NR | NR | anxiety and depression (HADS only associated with disordered eating in Caucasian sample |
| Wardle & Marsland (1990)  (94) | UK | Cross-sectional survey | Non-clinical | Dietary restraint (DEBQ) | 846 | 439F/407M (52% female) | R: 11-18 | *Physical appearance:* Asian/Oriental 20% Black 16% White Caucasian 63% | NR | *Determined by school:* High SES 40% Medium SES 26% Low SES 34% | NR |
| Bryant-Waugh & Lask (1991)  (95) | UK | Case series | Clinical | *DSM-III and ICD-9 diagnostic criteria:* AN (n = 4) | 4 | 4F (100% female) | R: 13-14 | *Participant or parental birthplace:* Bangladeshi (n = 1) Indian (n = 3) | NR | NR | depression (n = 1); depression (symptoms) (n = 2); anxiety (symptoms) (n = 1) |
| Mumford et al. (1991)  (96) | UK | Cross-sectional survey | Non-clinical | Disordered eating (EAT-26) | 559 | 559F (100% female) | Whole sample: R: 14-16  Asian sample:  M: 15.1, SD: 1.6 Caucasian sample:  M: 14.9, SD: 1.0 | Caucasian 64% South Asian 36% | Asian sample:  Muslim 88%, Sikh 9%, Hindu 3% Caucasian sample: Christian, Jewish | NR | NR |
| Wardle et al. (1993)  (97) | UK | Cross-sectional survey | Non-clinical | Dietary restraint (DEBQ) | 274 | 274F (100% female) | Whole sample: R: 14-22 years Asian sample: M: 14.9, SD: 0.81 (school sample); M: 20.4, SD: 2.4 (college sample) White sample: M 15.3, SD: 1.08 (school sample); M: 21.0, SD: 3.8 (college sample) | *Self-report:* Asian 46% White 54% | NR | NR | NR |
| Ballard et al. (1993)  (98) | UK | Case study | Clinical | NR | 1 | 1F | 6 | Asian | NR | NR | Ethylmalonic aciduria |
| Ahmad et al. (1994a)  (99) | UK | Cross-sectional survey | Non-clinical | Disordered eating (EAT-26) | 186 | 186F (100% female) | Whole sample: R: 14-15 Asian sample:  M: 14.9, SD: 0.69 Caucasian sample:  M: 14.9, SD: 0.73 | Asian (Indian subcontinent) 38% Caucasian 62% | NR | NR | NR |
| Ahmad et al. (1994b)  (100) | UK | Cross-sectional survey | Non-clinical | Disordered eating (EAT-26) | 354 | 186F/168M (53% female) | Girls: M: 14.9 Boys: M: 15.0 | *Self-report:* Asian (Indian subcontinent) 37% Caucasian 63% | Asian sample:  Muslim 77% Hindu 23% | NR | NR |
| Furnham & Patel (1994)  (101) | UK | Cross-sectional survey | Non-clinical | Disordered eating (EAT-26) Binge eating (BEQ) | 96 | 96F (100% female) | R: 12-18 M: 15.0 SD: 2.39 | *Parental ethnicity:* Asian (Bangladeshi, Indian, Pakistani) 77% Caucasian 23% | agnostic 3% Anglican 26% atheist 10% Catholic 2% Hindu 10% Jewish 14% Muslim 13% other Protestant 22% | *Determined by father's occupation:* High SES 39% Middle SES 37% Lower SES 24% | NR |
| Chapman & Procopio (1995)  (102) | UK | Case study | Clinical | *DSM-III-R diagnostic criteria:* AN | 1 | 1M | 15 | *Parental ethnicity:* Chinese | NR | NR | NR |
| Soomro et al. (1995)  (103) | UK | Retrospective case review | Clinical | DSM-III-R diagnostic criteria:  non-White sample: AN 88%, partial syndrome AN 12% White sample: AN 100% | 985 | 91% female | non-White patients:  M: 21.1, SD: 4.2 White patients:  M: 23.6, SD: 8.3 | *Self-report and physical appearance:* non-White (Asian, Afro-Caribbean, mixed race, other ethnicity) 4% White 96% | NR | *RGSC:*  non-White Sample: Class I 27%, Class II 39%, Class III 30%, Class IV 3% White Sample: Class I 33%, Class 2 34%, Class III 23%, Class IV 9%, Class V 1% | NR |
| Hill & Bhatti (1995)  (104) | UK | Cross-sectional survey | Non-clinical | Dietary restraint (DEBQ) | 97 | 97F (100% female) | 9 | Asian 57% Caucasian 43% | Asian sample: Muslim 93% | NR | NR |
| McCourt & Waller (1995)  (105) | UK | Cross-sectional survey | Non-clinical | Disordered eating (EAT-26) | 336 | 336F (100% female) | R: 12-16 | *Self-report:* Asian (Indian subcontinent) 53% Caucasian 47% | Asian sample:  Muslim 92% Hindu 8% | NR | NR |
| Waller et al. (1995)  (106) | UK | Cross-sectional survey | Non-clinical | Bulimic symptoms (BITE) | 260 | 260F (100% female) | Whole sample: R: 14-15 Asian sample:  M: 14.5, SD: 0.54 Caucasian sample:  M: 14.5, SD: 0.60 | *Self-report:* Asian (Indian subcontinent) 59% Caucasian 41% | Asian sample: predominantly Muslim | NR | NR |
| Reiss (1996)  (107) | UK | Two-phase prevalence study | Non-clinical | Bulimic symptoms (BITE) DSM-III-R diagnostic criteria | 418 | 418F (100% female) | African-Caribbean sample:  M: 26.0, SD: 5.0 White sample:  M: 25.3, SD: 5.8 | *Self-report and visual/auditory appraisal:*  African 9% African-Caribbean (including Guyanan) 33% Asian (including East African Asian, Indian, Pakistani, Sri Lankan) 2% Asian Caribbean 1% Cypriot (Greek & Turkish) 4% Mauritain 2% other ethnicity/mixed race 5% White 46% | NR | *RGSC (patient or partner occupation):* African-Caribbean sample: 81.9% in non-manual social classes (Class I-IIIN) White sample: 76% in non-manual social classes (Class I-IIIN) | NR |
| Ratan et al. (1998)  (108) | UK | Retrospective case review | Clinical | *ICD-10 diagnostic criteria:* AN (n = 3) AAN (n = 3) BN (n = 10) ABN (n = 2) Vomiting associated with other psychological disturbances (n = 2) Other ED (n = 1) No ED (n = 3) | 24 | 23F/1M (96% female) | R: 18-39 | *Personal/family background:* Asian (Bangladeshi, Pakistani, Indian) 100% | NR | *OPCS (patient occupation):* Class II 10% Class III 24% Class IV 19% Class V 5% Students 43% | Self-harm (n = 6) |
| Button et al. (1998)  (109) | UK | Cross-sectional survey | Non-clinical | Disordered eating (EAT-26) | 235 | 235F (100% female) | R: 18-27 | *Self-report (census categories):* Asian 25.8% (African background 13.3%; Indian background 83.6%) Black 11% Caucasian 59.8% other ethnicity 3.4% | Asian sample: Muslim 30% Sikh 43% Hindu 22% | NR | NR |
| Ogden & Elder (1998)  (110) | UK | Cross-sectional survey | Non-clinical | Dietary restraint (DEBQ) | 100 (50 mother-daughter dyads) | Daughters:  50 F (100% female) Mothers:  50F (100% female) | Daughters:  R: 18-26, M: 20.02, SD: 1.75 Mothers:  R: 39-60, M: 49.74, SD: 4.98 | *Self-report:* Asian 50% (Indian n = 32; Pakistani n = 2; Sri Lankan n = 6; other Asian n = 10) White 50% | NR | Middle to upper class. Controlled for SES by recruiting students from medical school. | NR |
| Ogden & Chanana (1998)  (111) | UK | Cross-sectional survey | Non-clinical | Dietary restraint (DEBQ) | 160 (40 family units; daughter, father, mother, sibling) | Daughters: 40F (100% female) Mothers:  40F (100% female) Fathers: 40M (100% male) Siblings: 26F (65% female) | Asian daughters:  M: 20.5, SD: 0.6 White daughters:  M: 20.9, SD: 0.7 | *Self-report:* Asian 50%  White 50% | NR | *Self-report and highest household education:*  Predominately upper middle to upper class. Asian and White families matched for social class. | NR |
| Furnham & Husain (1999)  (112) | UK | Cross-sectional survey | Non-clinical | Disordered eating (EAT-26) | 137 | 137F (100% female) | Whole sample: R: 18-21 Asian sample:  M: 22.13, SD: 3.48 Caucasian sample:  M: 21.15, SD: 2.18 | *Parental birth place:* Asian (Indian, Pakistani) 40% Caucasian 60% | Asian sample: predominantly Muslim | *RGSC (parent occupation):* Asian sample: Professional 38%, Skilled 46%, Unskilled 11%, Unemployed 7% White sample: Professional 34%, Skilled 50%, Unskilled 9%, Unemployed 6% | NR |
| Mumford & Choudry (2000)  (113) | UK | Two-phase prevalence study | Non-clinical | Disordered eating (EAT-26) DSM-III-R diagnostic criteria | 104 | 104F (100% female) | British South Asian sample:  M: 29.6, SD: 11.4 Pakistani sample:  M: 23.6, SD: 6.7 White British sample:  M: 36.1, SD: 11.8 | British South Asian 28% (Gujrati n = 4; Pathan n = 1; Punjabi n = 22; other Asian n = 2) Pakistani 34% White British 38% | NR | British Asian sample: mostly middle class Pakistani sample: predominantly affluent social backgrounds White British sample: less affluent social backgrounds | NR |
| Furnham & Adam-Saib (2001)  (114) | UK | Cross-sectional survey | Non-clinical | Disordered eating (EAT-26) | 168 | 168F (100% female) | Whole Sample: R: 15-17, M: 15.55, SD: 0.58 Bengali sample: M: 15.47, SD: 0.51 Indian sample = 15.60, SD: 0.71 Pakistani sample = 15.48, SD: 0.51 White sample = 15.63, SD: 0.58 | *Parental birth place:* Bengali 23% Indian 24% Pakistani 26% White 27% | Bengali sample: Muslim 100% Indian sample: Muslim 40%, Hindu 30%, Sikh 30%  Pakistani sample: Muslim 100% White sample: Christian 70%, Jewish 30% | NR | NR |
| Mujtaba & Furnham (2001)  (115) | UK | Mixed-method study | Non-clinical | Disordered eating (EAT-26) | 348 | 348F (100% female) | British South Asian sample: M: 20.0, SD: 3.14 White Caucasian sample: M: 20.4, SD: 3.02 Pakistani sample: M: 19.6, SD: 1.58 | British Asian 34% British Caucasian 33% Pakistani 33% | British Asian sample:  Muslim 100% | *Parent occupation:*  British Asian sample:  0% fathers & 0% mothers professional  British Caucasian sample:  59% fathers & 33% mothers professional Pakistani sample:  49% fathers & 10% mothers professional | NR |
| Thomas et al. (2002)  (116) | UK | Cross-sectional survey | Non-clinical | Disordered eating (EAT-26) | 722 | 387F/335M (54% female) | R: 11-16 | *Self-report:* Asian/Muslim 18% Black/African-Caribbean 16% mixed race 3% other ethnicity 4% White 60% | NR | *RGSC (parent occupation):* 82% of families with at least one family member employed | Depression (Angold Mood and Feelings Scale): depressed mood independently associated with high EAT-26 scores |
| Bhugra & Bhui (2003)  (117) | UK | Two-phase prevalence study | Non-clinical | Bulimic symptoms (BITE) DSM-III-R diagnostic criteria | 266 | 127F/139M (48% female) | Asian sample:  M: 14.11, SD: 0.64 African-Caribbean sample:  M: 14.10, SD: 0.71 'Other' ethnicity sample: M: 13.11, SD: 0.62 White sample:  M: 14.09, SD: 0.65 | *Self-report (OPCS categories):* African-Caribbean 19% Asian (Bangladeshi, Indian, Pakistani, Sri Lankan) 20% other ethnicity 11% White 50% | NR | *Determined by father's occupation:*  predominately Class III (Asian sample 46%, Black sample 40%, 'other' ethnicity sample 53%, White sample 50%) | NR |
| Tareen et al. (2005)  (118) | UK | Retrospective case review | Clinical | South Asian sample: AN (n= 6), AAN (N = 8) White sample: AN (n = 14) | 28 | 28F (100% female) | Whole sample:  R: 11-18 South Asian sample:  M: 15.07 White British sample:  M: 14.97 | *Self-report (census categories):* British South Asian (Bangladeshi, Indian, Pakistani) 50% White British 50% | NR | British South Asian sample: mainly social class III or V White British sample: mainly social class I or II | Other psychiatric conditions (South Asian/White):  ICD-10 depressive disorder (n = 3/7); depression (symptoms) (n = 11/6) |
| Currin et al. (2007)  (119) | UK | Vignette study | Clinicians | Possible diagnosis of AN, BN, BED or EDNOS given to vignettes | 82 | 44F/38M (54% female) | NR | NR; Vignette manipulated to be Afro-Caribbean or White patient | NR | NR | NR |
| Dave (2008)*  (120) | UK | Qualitative study | Clinical | *DSM-IV diagnostic criteria:* South Asian sample: EDNOS (n = 5) White sample: BN (n = 4), ENDOS (n = 1) | 10 | 10F (100% female) | Whole sample: 19-52 South Asian sample:  M: 36.4 White sample:  M:24.4 | *Self-report:* South Asian (Bangladeshi, Indian, Pakistani) 50% White British 50% | NR | NR | NR |
| Waller et al. (2009)  (121) | UK | Retrospective case review | Clinical | *DSM-IV diagnostic criteria:* non-White sample: AN 22%, BN 54%, EDNOS 19%, No ED 5% White sample: AN 28%, BN 34%, EDNOS 27%, No ED 11% | 648 | 567F/81M (88% female) | White sample:  M: 28.3, SD: 8.71 non-White sample:  M: 27.8, SD: 6.17 | *Self-report (census categories):* Black 6% South Asian 4% other ethnicity 2% White 88% | NR | NR | NR |
| Abbas et al. (2010)  (122) | UK | Retrospective case review | Clinical | *DSM-IV diagnostic criteria:* South Asian sample: AN 10%, BN 27%, EDNOS 45%, No ED 15%, ED diagnosis missing 3% non-Asian sample: AN 17%, BN 27%, EDNOS 46%, No ED 9%, ED diagnosis missing 2% | 2070 | 1970F/100M (95% female) | South Asian sample:  M: 23.4, SD: 6.08 non-Asian sample:  M: 27.1, SD: 8.86 | *Self-report and patient name:* South Asian 5% non-Asian 95% | NR | NR | NR |
| Hoque (2011)*  (123) | UK | Qualitative Study | Clinical | AN (n = 2) BN (n = 2) EDNOS (n = 2) | 6 | 6F (100% female) | R: 17-29 | Asian (Indian, Pakistani) 100% | Muslim 67% Hindu 33% | NR | NR |
| Chowbey et al. (2012)  (124) | UK | Qualitative Study | Mixed: non-clinical, relatives and clinicians | Any ED | 42 (24 community members; 3 relatives, 15 key informants) | Community members: F/M Relatives: NR Key informants: NR | Community members:  R: 18-24 Relatives: NR Key Informants: NR | *Self-report:* Community members: Somali or Bangladeshi Relatives: Indian or Pakistani Key informants: NR, experience of working with minority ethnic communities | NR | NR | NR |
| Dogra et al. (2013)  (125) | UK | Cross-sectional survey | Non-clinical | Disordered eating (SCOFF) | 2900 | 1610F/1290M (56% female) | R: 13-15  M: 13.97 SD: 0.7 | *Self-report:* Indian 38% White 14% | Asian sample: Muslim 27%, Hindu 57% White sample: Christian 42%, atheist/agnostic 40% | NR | NR |
| Solmi et al. (2014)  (126) | UK | Population survey (SELCoH I) | Non-clinical | Disordered eating (SCOFF) | 1698 | 931F/767M (57% female) | R: 16-90 M: 36.9 SD: 16.8 | *Self-report:* Asian (Bangladeshi, Indian, Pakistani, Chinese) 4% Black (Black African, Black Caribbean) 22% mixed/other ethnicity 12%  White 62% Missing data (n = 55) | NR | *Educational attainment:* Degree level or above 42% GSCEs/A-Levels 53% No Qualifications 13% | Alcohol use (AUDIT); Anxiety (CIS-R); Depression (CIS-R); Personality disorder (SAPAS); PTSD (PC-PTSD); Substance use; Suicidal ideation and/or attempt |
| Nazir (2015)*  (127) | UK | Qualitative Study | Mixed: clinical, relatives and clinicians | AN (n = 7) AN & BN (n = 3) Disordered eating (EDE-Q) | 25 (10 ED patients; 7 relatives; 18 clinicians) | ED patients: 10F (100% female) Relatives:  7F (100% female) Clinicians: 13F/5M (72% female) | ED patients: R: 18-64  Relatives: NR Clinicians: NR | ED patients: Bengali (n = 2), Indian (n = 3), Pakistani (n = 5) Relatives: Bengali (n = 3), Pakistani (n = 4) Clinicians: Afro-Caribbean (n = 1), Bengali (n = 1), Caucasian (n = 4), Indian (n = 1), Pakistani (n = 11); experience working with South Asian women with EDs | NR | NR | NR |
| Solmi et al. (2016)  (10) | UK | Two-phase prevalence study (SELCoH II) | Non-clinical | Disordered eating (SCOFF) DSM-V diagnostic criteria | 145 | 109F/36M (75% female) | R: 16-90 | *Self-report:* Asian (Bangladeshi, Indian, Pakistani, Chinese) 3% Black (Black African, Black Caribbean) 30% mixed/other ethnicity 11% White 57% | NR | *Educational attainment:* University degree 50% GSCEs/A-Levels 53% No qualifications 11% | Alcohol use (AUDIT); Anxiety (CIS-R); Depression (CIS-R); Personality disorder (SAPAS); PTSD (PC-PTSD); Substance use; Suicidal ideation and/or attempt |
| Swami (2016)  (128) | UK | Longitudinal survey | Non-clinical | Disordered eating (EDI-3) | 98 | 98F (100% female) | R: 18-22 | Malaysian 100% | NR | NR | NR |
| Chaudary*  (2017)  (129) | UK | Vignette study | Clinicians | Possible diagnosis of AN, BN or BED given to vignette | 156 | 121F (78% female) | R: 24-65 | Asian 3.2%  Black 1.9%  mixed 1.9%  other ethnicity 1.3% (n = 1 Berber, n = 1 North African)  White 91.7%  Vignette manipulated to be South Asian or White | NR | NR | NR |
| Wales et al. (2017)  (130) | UK | Qualitative study | Mixed: non-clinical and clinicians | Any ED | 44 (16 clinicians; 28 community members) | Clinicians: 12F/4M (75% female) Community members: 23F/5M (82% female) | Clinicians: NR Community members: 18-65 | S*elf-report:* Community members: South Asian Clinicians: NR; experience working with South Asian patients | NR | NR | NR |
| Persuad (2017)*  (131) | UK | Qualitative study | Clinical | NR | 9 | 9F (100% female) | R: 17-48 | *Self-report:* Indian (n = 1) Jamaican (n = 1) Pakistani (n =1) multiple ethnic groups (n = 1 Jamaican & Irish; n = 1 Somalian & Arabic, n = 1 African & Bermudian, n = 1 Jamaican & South African, n = 1 Mauritian & Irish, n = 1 Japanese & British) | Buddhist 22% Catholic 11% Muslim 22% Seventh Day Adventist 11% Sikh 11% Not reported 22% | NR | NR |
| Petkova et al. (2019)  (132) | UK | Observational surveillance study | Clinical | *DSM-5 diagnostic criteria:* AN 100% | 305 | 279F/26M (91% female) | R: 8-17 | Black Caribbean (n = 2) Chinese (n = 1) Indian (n = 3) Pakistani (n = 2) Bangladeshi (n = 1) other Asian (n = 4) White (n = 274) White & Asian (n = 6) White & Black Caribbean (n = 2) White and Black African (n = 1) other mixed (n = 1) ethnicity unknown (n = 2) | NR | NR | NR |
| Channa et al. (2019)  (133) | UK | Qualitative study | Clinical | *DSM-5 diagnostic criteria:* BN | 1 | 1F | Early 20s | South Asian (Indian) | NR | NR | NR |
| Kanakam (2022)  (134) | UK | Qualitative study | Clinicians | Any ED | 12 | 11F/1M (92% female) | NR | White British/White other 67% Minority ethnic background 33% Experience working with minority ethnic women with EDs | NR | NR | NR |
| Cooper et al. (2023)  (135) | UK | Secondary data analysis | Clinical | NR | 12,361,554 | 6,137,121F/6,224,433M (50% female) | R: 0-70+ Median: 39.5 IQR: 22.5-58.5 | Asian 10% Black 5% Mixed 2% other ethnicity (including Chinese, Middle Eastern & Pacific) 2% White 81% Data missing (n = 2,529,141) | NR | *British IMD:* 1st quintile 19.7% 2nd quintile 19.5% 3rd quintile 18.9% 4th quintile 20.3% 5th quintile 19.2% missing data 2.5% | NR |
| Dalton et al. (2024)  (136) | UK | Observational study | Clinical | Whole Sample: BN (n = 51), BED (n = 67), OSFED (n = 12) | 130 | 109F/21M (84% female) | M: 34.03 SD: 11.24 | *Self-report:* Black/Black British 14% Asian/Asian British 5% mixed or multiple ethnic groups 6% other ethnicity 4% White 71% | NR | NR | anxiety 19%; depression 38%; other mental health disorder 10%; neurodevelopmental disorder 5%; type II diabetes 9%; other physical health condition 11%; none listed 40% |

^a.^ Ethnicity is reported as described in original research paper.

Abbreviations: AN – Anorexia Nervosa; ADHD – Attention Deficit Hyperactivity Disorder; ASCO – Australian Standard Classification of Occupations; AUDIT – Alcohol Use Disorders Identification Test; BED – Binge Eating Disorder; BEQ – Binge Eating Questionnaire; BITE – Bulimic Inventory Test, Edinburgh; BN – Bulimia Nervosa; BULIT-R – Bulimia Test Revised; CI – Confidence interval; CIS-R – Clinical Interview Schedule Revised; DEBQ – Dutch Eating Behaviour Questionnaire; DSM – Diagnostic and Statistical Manual; EAT-26, EAT-40 – Eating Attitudes Test; ED-15 – Eating Disorder-15; EDE – Eating Disorder Examination; EDI – Eating Disorder Inventory; EDNOS – Eating Disorder Not Otherwise Specified; HADS – Hospital Anxiety and Depression Scale; ICD – International Classification of Disorders, IMD – Index of Multiple Deprivation; IQR - Interquartile range; NEQ – Night Eating Questionnaire; NES – Night Eating SyndromeNZDep2001 – New Zealand 2001 Deprivation Index; OPCS – Occupational Population Classification System; OSFED – Other Specified Feeding or Eating Disorder; PTSD – Post Traumatic Stress Disorder; PTSD-PC – Primary Care Post Traumatic Stress Disorder Screen; RGSC – Registrar’s General Social Class Classification System; SAPAS; Structured Assessment of Personality Abbreviated Scale; SEED – Short Evaluation of Eating Disorders; SES – Socioeconomic Status.

* Thesis

## **Table S2** Case studies and case series

| **First Author & Year of Publication** | **Country** | **Aim/Focus** | **Key Data Collected** | **Summary of findings** |
| --- | --- | --- | --- | --- |
| Birmingham and Sidhu (2007)  (67) | Canada | To describe a case of AN in a Southeast Asian woman who chose alternative therapy over conventional treatment. | Case history and description | Typical presentation of AN symptoms. Upon discharge patient chose to continue with zen therapy over conventional treatment. Responded well to treatment during admission and food intake and mood improved. |
| Cheung & Wilder-Smith (1995)  (74) | New Zealand | To describe a case of AN and schizophrenia in a Chinese man. | Case history and description | Presentation of AN symptoms typical and comparable to other men patients. Unlike Chinese women with AN fear of fatness and distorted body image were present. |
| Thomas & Szmukler (1985)  (85) | UK | To describe three cases of EDs in Afro-Caribbean patients. | Case history and description | Clinical characteristics of were typical and comparable to White patients. |
| Lacey & Dolan (1988)  (89) | UK | To describe the sociodemographic and clinical characteristics of non-White BN patients. | Case history and description, referral rates | Clinical characteristics of non-White patients comparable to White patients. Compared to White patients non-White patients were more likely to come from broken homes. Referral rates for non-White women lower than expected based on local catchment area. Poor treatment response for non-White women, only N = 1 showed sustained improvement. |
| Fahy et al. (1988)  (90) | UK | To describe a case of AN in an African woman following torture. | Case history and description, EAT-26 | Symptoms consistent with a diagnosis of atypical AN. Onset of ED coincided with torture. EAT-26 scores of 10, 13 and 4 when administered routinely at 2-month intervals. Responded well to treatment and achieved weight restoration. |
| Ford & Dolan (1989)  (91) | UK | To describe a case of BN in a British Sikh woman. | Case history and description | Unusually early onset of BN which coincided with development of asthma. Clinical characteristics and symptoms otherwise considered typical. History of multiimpulsivity, abuse and pre-morbid obesity, dieting and AN. BN associated with subsequent multiple miscarriages. Family and cultural stressors (e.g., family conflict, rejection by Sikh community) may have played a role in the development of BN. Poor engagement with treatment. |
| Bhadrinath (1990)  (92) | UK | To describe three cases of AN in Asian adolescents. | Case history and description | Clinical characteristics considered typical. Ramadan associated with worsening of condition in Muslim patients. Attempts of family therapy abandoned in N = 1 due to parental reluctance to engage. N = 2 discharged prematurely. |
| Bryant-Waugh & Lask (1991)  (95) | UK | To describe four cases of AN in Asian children. | Case history and description, referral rates | Clinical characteristics considered typical. Sociocultural conflict identified as a possible contributing factor in the development of AN in all cases. Addressing family issues and cross-cultural conflict was a central of treatment. Parents of N = 1 patient refused to engage in family therapy. N = 2 discharged prematurely due to poor engagement. Of 30 referrals for girls with AN over a 3-year period, 13% (N = 4) were of Asian origin. |
| Ballard et al. (1993)  (98) | UK | To describe a case of an ED in an Asian girl. | Case history and description | No evidence of body image disturbance or fear of fatness. Co-morbid ethylmalonic aciduria triggered and maintained ED symptoms. Family dynamic and conflict also thought to be maintaining factors. Slow progress but exhibited weight gain over a 1-month period. Poor family compliance with prescribed eating programme. |
| Chapman & Procopio (1995)  (102) | UK | To describe the case of AN in a Chinese boy. | Case description and history. | Initially presented with physical complaints which delayed diagnosis. Clinical presentation otherwise considered typical. Parents initially declined in-patient admission and sought alternative treatments and explanations of weight loss. Once admitted made slow progress and gained weight. |

Abbreviations: AN – Anorexia Nervosa; BN – Bulimia Nervosa; EAT – Eating Attitudes Test; ED – Eating Disorder; UK – United Kingdom

## **Table S3** Qualitative studies

| **First Author & Year of Publication** | **Country** | **Aim/Focus** | **Data Collection & Method** | **Summary of findings** |
| --- | --- | --- | --- | --- |
| Mustafa et al. (2016)  (73) | Canada | To explore second-generation Canadian South Asian women’s experiences of EDs and identify cultural factors which may influence the development of EDs. | Semi-structured interviews Phenomenological approach | Cultural conflict, stigma towards mental health in the South Asian community and pressure to uphold cultural and familial expectations were found to contribute to the development and maintenance of EDs and contributed to silence and shame around the ED which delayed help-seeking. |
| Clark et al. (2023)  (84) | New Zealand | To explore the lived experiences of Māori with EDs and their family/support system to identify barriers and facilitators to accessing specialist ED services. | Semi-structured interviews Thematic Analysis | Systemic barriers identified included idiosyncratic use of assessment methods, variability in quality of service provisions, inaccessible service locations, and limited number of available beds. Social barriers included dominance of a Western/European narrative of EDs within services, stereotypes around who is affected by EDs and what causes EDs, shame, stigma and discrimination. Social facilitators included self-advocacy, good support systems, health promotion in the media and mental health literacy. |
| Dave (2008)*  (120) | UK | To investigate the similarities and differences in factors contributing to the onset of EDs and influencing access to treatment in South Asian and White ED patients. | Semi-structured interviews | Some similarities in the factors associated with the development of EDs (e.g., adverse childhood experiences, negative self-image). Cultural factors contributing to the maintenance of EDs and difficulties in accessing family support and treatment specific to South Asian participants also highlighted (e.g., silence and stigma around mental health, family rules and expectations, lack of knowledge of eating disorders, fear of being ostracised). |
| Hoque (2011)*  (123) | UK | To explore the journey into and experience of ED treatment for South Asian girls and women. | Semi-structured interviews Interpretative phenomenological analysis | Parental response and cultural values of the South Asian community were integral to the experience of having an ED and accessing treatment. Most parents refused to acknowledge their daughters ED due to associated shame and stigma. Parents also feared 'outsider intervention' and specialist ED treatment was often only sought as a last resort. The role of treatment and response from clinicians was spoken about positively and contrasted against response of parents. |
| Chowbey et al. (2012)  (124) | UK | To identify which areas need to be addressed to allow for the early detection and treatment of EDs in minority ethnic populations. | Focus Groups (N = 2 with men from the community, N = 2 with women from the members) using a vignette of an AN case to guide discussion  In-depth interviews with relatives and key informants | Low awareness of EDs, cultural norms and ideals relating to body image and food, religious practices, family structures and functioning, stigma and poor past experiences with services were found to influence the recognition of and response to EDs. |
| Nazir (2015)*  (127) | UK | To explore factors influencing the development and maintenance of EDs and help-seeking in South Asian women. | Semi-structured interviews Thematic Analysis | Onset and maintenance of EDs related to familial and cultural conflict, with conflict revolving around marriage, education, career choices, socialising and gendered roles and expectations. Barriers to seeking treatment included a lack of familial support and understanding, fear of bringing shame to the family, stigma of mental health, confidentiality concerns and issues with service provision (e.g., lack of resources, training and knowledge). |
| Wales et al. (2017)  (130) | UK | To understand why South Asian patients are underrepresented in referrals to specialist ED services and identify barriers to help-seeking for EDs within this population. | Focus Groups (N = 1 with clinicians, N = 6 with community members) | Cultural norms and ideals around body image and food, lack of knowledge about EDs and their potential severity, family structures and functioning, stigma towards mental health, privacy and confidentiality concerns were found to influence the recognition of EDs and delay help-seeking. |
| Persuad (2017)*  (131) | UK | To explore how minoritized girls and women construct narratives of recovering from EDs. | Narrative interviews | Minority ethnic girls and women with EDs may present similarly to their White counterparts but pathways to the condition may differ. Factors including migration, racism, colonisation, slavery, trauma, loss, bereavement, famine, war, starvation, the role of food, gender roles, family understanding of mental ill health and social change should be considered in assessment and treatment. Help-seeking among minoritized girls and women is delayed and there are issues with accessing treatment. Minority ethnic girls and women are positive about their recovering from EDs and co-existing within individualistic and collectivist cultures may assist in this recovery. |
| Channa et al. (2019)  (133) | UK | To explore the factors contributing to the development of BN in a British Indian woman. | Semi-structured interview Interpretative phenomenological analysis | Factors contributing to development and maintenance of BN included body image concerns, poor self-esteem, perfectionism, emotional eating, categorisation of food as 'good' and 'bad', loneliness, conflict between university and South Asian cultures. Lack of parental support, silence, cultural stigma and shame towards mental health and associated treatment, lack of knowledge and awareness of EDs in South Asian culture led to delayed help-seeking |
| Kanakam (2022)  (134) | UK | To explore therapists perspectives on how minority ethnic girls and women access specialist ED services and their experiences of working with minority ethnic girls and women. | Semi-structured interviews Thematic analysis | Reduced family support, shame and cultural differences in clinical presentation and understanding of EDs were thought to delay help-seeking and complicate pathways into specialist services. Therapists expressed a desire to offer diverse and culturally adapted therapy but felt restricted by clinical guidelines, service structures and management and having limited time, knowledge and resources. |

Abbreviations: AN – Anorexia Nervosa; ED – Eating Disorder; BN – Bulimia Nervosa,

* = Thesis

## **Table S4** Retrospective case note review

| **First Author & Year of Publication** | **Country** | **Aim/Focus** | **Key Data Collected** | **Summary of findings** |
| --- | --- | --- | --- | --- |
| Rieger et al. (2000)  (52) | Australia | To examine cross-cultural assumptions regarding weight concerns in AN. | Medical record data regarding weight concerns | All patients presented with egosyntonic emaciation. Motivations for egosyntonic weight loss varied and included ‘fat phobia’ and seeking a sense of control. ED diagnosis preceded residence in Australia for N = 6. |
| Holden & Robinson (1988)  (88) | UK | To compare sociodemographic and clinical characteristics of Black ED patients referred to a London hospital between 1979 and 1985 with a control group of White patients. | Medical records data, referral rates | 457 White patients vs. 13 Black patients referred during time period. Broadly similar sociodemographic and clinical characteristics between ethnic groups. Black patients significantly more likely to be diagnosed with BN than White patient (85% vs. 48%). Black patients more likely to have experienced parental divorce/separation and premorbid obesity prior to diagnosis and to be referred from emergency psychiatric services than primary care services. |
| Soomro et al. (1995)  (103) | UK | To compare sociodemographic and clinical characteristics and rate of presentation of White and non-White AN patients referred between 1960 and 1993. | Medical record data, referral rates | Approximately 6-7% of all referrals were non-White. Similarity of clinical and social characteristics between non-White and White patients. Compared to White women, non-White women presented at younger ages and were less likely to report experiencing sensitivity to fatness (12% vs. 5%). |
| Ratan et al. (1998)  (108) | UK | To examine the number and clinical characteristics of referrals for Asian patients to an adult ED service in Leicester between 1984 and 1994. | Medical record data, referral rates | Asian patients less likely to be referred than non-Asian patients. Asian patients closely resembled non-Asian counterparts in age and clinical characteristics. 80% of women AN patients and 33% of bulimic patients reported “cultural tension” prior to onset of ED. |
| Tareen et al. (2005)  (118) | UK | To investigate clinical features of British South Asian girls who presented to CAMHS services in West London from 1922 onwards. | Medical record data | Compared to White British Counterparts, South Asian girls presented more frequently with loss of appetite and less frequently with fat phobia, exercise to control weight and preoccupation with food and weight. South Asian girls less likely to receive a diagnosis of AN. |
| Waller et al. (2009)  (121) | UK | To examine the influence of ethnicity on referral rate, diagnosis and treatment offered for referrals to an adult ED service in London between 2002 and 2003. | Medical record data, referral rates | In relation to the local population, non-White patients were less likely to be referred. White patients overrepresented by 25%, whilst Asian and Black patients were under presented by 40% and 70% respectively. Non-White patients more likely to be diagnosed with BN and less likely to have no ED diagnosis. No significant differences in treatment offered across ethnic groups. |
| Abbas et al. (2010)  (122) | UK | To compare the referral rate, diagnosis, clinical presentation and treatment of South Asian and non-Asian patients referred to an adult ED service in Leicester between 1991 and 2005. | Medical record data, referral rates | South Asian were underrepresented, only 4.5% of women patients were South Asian compared to 13.8% of the local population. No significant differences in distribution of diagnoses between ethnic groups. However, a non-significant trend where South Asian patients were less likely to be diagnosed with AN was found. Women South Asian BN and EDNOS patients resembled matched non-Asian counterparts in terms of clinical features and treatment offered. South Asian patients were on average significantly younger. |

Abbreviations: AN – Anorexia Nervosa; BN – Bulimia Nervosa; CAMHS – Child and Adolescent Mental Health Service; ED – Eating Disorder, ENDOS – Eating Disorder Not Otherwise Specified; UK – United Kingdom

## **Table S5** Secondary data analysis

| **First Author & Year of Publication** | **Country** | **Aim/Focus** | **Data Source** | **Summary of findings** |
| --- | --- | --- | --- | --- |
| Hay & Carriage (2012)  (60) | Australia | To investigate the prevalence of ED behaviours and psychopathology in older adolescent and adult Indigenous Australians. | South Australia Health Omnibus Surveys 2005 and 2008. Interview data on sociodemographic characteristics, physical health and disordered eating behaviours (EDE). | Greater levels of disordered eating in Indigenous sample. In 2005 when controlling for age, income and gender, presence of weekly objective binge eating (OR = 2.5, 95% CI 1.4-4.4) and moderate to high levels of overvaluation of body weight/shape (OR = 2.1. 95% CI 1.3-3.3) was significantly greater for Indigenous group compared to non-Indigenous group. No differences in frequency of ED symptoms found in 2008. |
| Mulders-Jones et al. (2017)  (61) | Australia | To investigate the association between Indigenous status and prevalence of ED features. | South Australia Health Omnibus Surveys 2008 and 2009. Interview data on sociodemographic characteristics, physical health and disordered eating behaviours (EDE). | No significant differences in the rate of disordered eating behaviours across Indigenous and non-Indigenous groups. Significantly greater levels of weight/shape overevaluation for participants who did not report Indigenous status compared to non-Indigenous participants. |
| Cheah et al. (2020)  (62) | Australia | To investigate the prevalence of EDs in first-generation migrants to Australia comparison with the Australian-born population. | South Australia Health Omnibus Surveys 2015 and 2016. Interview data on sociodemographic characteristics, physical health and disordered eating behaviours (EDE). | 3-month prevalence of any ED was significantly lower in first-generation migrants (4.5%, 95% CI 3.6-5.6) compared to Australian-born population (6.4%, 95% CI 5.7-7.2). Those born in Africa (11%, 95% CI 6.1-19.1) had higher prevalence of EDs than those born in Asia (4.0%, 95% CI 2.7-5.8). |
| Burt et al. (2020a)  (63) | Australia | To estimate the prevalence of DSM-5 EDs among First Australians adults and compare clinical features and health related QoL to non-Indigenous Australians. | South Australia Health Omnibus Surveys 2015 and 2016. Interview data on sociodemographic characteristics, physical health, health related QoL and disordered eating behaviours (EDE). | Prevalence of any ED for First Australian adults (27%, 95% CI 19.1-37.0) was significantly higher than for non-Indigenous adults. UFED characterised by recurrent binge eating was the most common diagnosis. Higher prevalence of EDs in First Australian adults was explained by this group being younger in age, having a higher BMI and poorer mental health. First Australian adults with an ED had higher levels shape/weight overvaluation compared to other ethnic groups. |
| Burt et al. (2020b)  (64) | Australia | To estimate the prevalence, demographic distribution and burden of DSM-5 EDs among First Australian adolescents compared to non-Indigenous adolescents. | EveryBODY studies data from self-report surveys assessing sociodemographic characteristics, BMI, Paediatric QoL Scale (PedsQL), EDE-Q and NEQ. | Prevalence of any ED for First Australian adolescents (28.6%, 95% CI, 23.8-33.9) was significantly higher than for non-Indigenous adolescents (21.7%, 95% CI 20.4-23.0), largely due to high prevalence of OSFED-NES which was associated with poorer psychosocial QoL. All other ED diagnoses equally prevalent across ethnic groups. |
| Lacey et al. (2020)  (83) | New Zealand | To describe the population undergoing specialist ED treatment and compare clinical characteristics and service use for Māori and non-Māori. | Programme for Integration of Mental Health Data (PRIMHD) dataset. Analysed all records for patients with an ED diagnosis seen face to face by New Zealand specialist mental health services between 2009 to 2016. | Based on prevalence estimates, rate of presentation to specialist mental health services for Māori with ED was lower than expected. Patterns of service use and hospital events was similar for Māori and non-Māori. Compared to non-Māori, Māori had lower prevalence of AN (41.5 vs. 27.8%) but higher prevalence of BN (18.1% vs. 24.3%) and EDNOS (33.9% vs. 43.3%) as well as greater psychiatric co-morbidity. |
| Cooper et al. (2023)  (135) | UK | To describe the prevalence and sociodemographic variation of mental health and physical health conditions. | Clinical Practice Datalink (CPRD) Aurum primary care database. Identified prevalent cases using disease specific clinical code lists. | Prevalence of most mental health conditions including EDs were typically twice as high in White patients compared to minority ethnic patients. Prevalence rate of any ED was 0.7% for White patients, 0.6% for mixed ethnicity patients and 0.3% for Asian, Black and other ethnicity patients. |

Abbreviations: AN – Anorexia Nervosa; BMI – Body Mass Index; CI – Confidence Interval; DSM – Diagnostic and Statistical Manual; ED – Eating Disorder; EDE – Eating Disorder Examination; EDNOS – Eating Disorder Not Otherwise Specified; NES – Night Eating Syndrome; NEQ – Night Eating Questionnaire; OR – Odds Ratio; OSFED – Other Specified Feeding or Eating Disorder; QoL – Quality of Life; UFED – Unspecified Feeding or Eating Disorder

## **Table S6** Cross-sectional and longitudinal studies

| **First Author & Year of Publication** | **Country** | **Aim/Focus** | **Data Collected** | **Summary of findings** |
| --- | --- | --- | --- | --- |
| Lake et al. (2000)  (51) | Australia | To investigate the influence of ethnic identify on eating pathology and body dissatisfaction. | Sociodemographic characteristics, EAT-26, Figure Rating Scale (FRS), Ethnicity Identity Scale (EIS) | No differences in eating attitudes found between traditional Hong Kong-born girls and women and Australian-born girls and women. Acculturation effect found whereby compared to Australian-born students, acculturated Hong Kong-born students had significantly lower disordered eating and body dissatisfaction than Australian-born girls and women. |
| Ball & Kennardy (2002)  (53) | Australia | To investigate the role of acculturation in the relationship between ethnicity and eating pathology. | Sociodemographic characteristics, length of residence, BMI, adapted EDE-S, body weight dissatisfaction and dieting. | Risk factors for EDs identified across a range of ethnic groups. Fewer Asian-born women reported a history of dieting. Binge eating and use of compensatory behaviours (e.g., vomiting, laxative abuse, diuretics) did not differ across ethnic groups. Strong acculturation effect and women who had spent longer living in Australia reported more disordered eating. |
| Humphry & Ricciardelli (2004)  (54) | Australia | To examine the influence of acculturation and culture clash in the development of disordered eating. | BMI, EAT-26, Ethnicity Identity Scale (EIS), Perceived Sociocultural Influences on Body Image and Body Change Questionnaire (PSIQ), Parental Bonding Instrument (PBI) and Adult Self Perception Profile (ASPP) | Low satisfaction with physical appearance, high levels of perceived parental overprotection and perceived pressure to lose weight from women friends predicted greater levels of disordered eating for all Chinese-Australian women. Other risk factors for disordered eating differed between acculturated and traditional women. Higher levels of perceived parental care predicted greater levels of disordered eating in women with a strong Chinese ethnic identity. Whilst higher levels of perceived pressure to lose weight from fathers and men friends predicted greater levels of disordered eating in highly accultured women. |
| Jennings et al. (2005)  (55) | Australia | To compare ED behaviours and psychopathology between Asian and Caucasian adolescent girls and to examine the influence of acculturation on ED behaviours and psychopathology in Asian girls. | Sociodemographic characteristics, BMI, EAT-26, EDI-2, EDI-SC and Acculturation Index. | Total EDI-2 score and EAT-26 Dieting subscale scores were significantly higher for Asian girls compared to Caucasian girls. N= 4 Asian girls and N= 2 Caucasian girls scored above EAT-26 (>20) and/or EDI-2 (> 14 on Drive For Thinness subscale) cut-off points. N=2 Asian girls but no Caucasian girls reported a history of binging, purging and use of diet pills. Less acculturated Asian girls had greater disordered eating compared to more acculturated Asian girls. |
| Wang et al. (2005)  (56) | Australia | To examine the influences of SES and ethnicity on body dissatisfaction and eating behaviours in children and adolescents. | Sociodemographic characteristics, EAT-26, EDI body dissatisfaction items and self-perception of body size. | No significant differences in prevalence of disordered eating behaviours between ethnic groups. 5.7% Caucasian Australian participants, 3.1% of Chinese or Vietnamese participants and 8.2% of Italian or Greek participants scored above EAT-26 cut-off (>20). |
| Jennings et al. (2006a)  (57) | Australia | To compare ED behaviours and psychopathology between Caucasian Australian, Asian Australian and Thai women university students. | Sociodemographic characteristics, BMI, EAT-26, EDI-2 & EDI-SC. | Total EAT-26 and EDI-2 scores were highest for Thai students. Compared to Caucasian Australian students, Australian Asian students had higher total EDI-2 scores, however there was no significant differences between total EAT-26 scores. 11.3% of Asian Australian students, 10.0% of Caucasian Australian students and 14.9% of Thai students scored above EAT-26 (>20) and/or EDI-2 (> 14 on Drive For Thinness subscale) cut-off scores. |
| Jennings et al. (2006b)  (58) | Australia | To compare ED behaviours and psychopathology between Asian and Caucasian women university students and to examine the influence of acculturation on ED behaviours and psychopathology in Asian students. | Sociodemographic characteristics, BMI, EAT-26, EDI-2, EDI-SC and Acculturation Index. | Compared to Caucasian Australian students, Australian Asian students had higher total EDI-2 scores, however there was no significant differences between total EAT-26 scores. 11.3% of Asian students and 10.0% of Caucasian Australian students scored above EAT-26 (>20) and/or EDI-2 (> 14 Drive For Thinness subscale) cut-off scores. A greater proportion of Caucasian students had a positive screen on EDI-SC and reported a history of binging, purging and unhealthy weight control behaviours. No effect of acculturation on disordered eating. |
| Soh et al. (2007)  (59) | Australia | To explore the role of acculturation and sociocultural factors in the relationship between ED psychopathology and ethnicity in girls and women with and without EDs living in Australia and Singapore. | Sociodemographic characteristics, BMI, EDE-Q ('Global', 'Eating Concern' and 'Dietary Restraint' scores), Vancouver Index of Acculturation (VIA) and Family Cohesion Scale (FACES II). | Similar levels of eating pathology across all cultural groups for girls and women with EDs. For girls and women without EDs (controls), Singaporean Chinese girls and women had greater ‘global’ EDE-Q scores than all other cultural groups and greater ‘dietary restraint’ scores than North European Australians and expatriates. Eating concern was not associated with cultural group overall or acculturation. Satisfaction with family functioning, SES, and education level were not associated with disordered eating. |
| Geller (1996)*  (65) | Canada | To determine the prevalence of eating pathology and examine its association with acculturation in Native Canadian girls and women. | Sociodemographic characteristics, BMI, BULIT-R, Figure Rating Scale (FRS), Multidimensional Body Self-Relations Questionnaires (MBSRQ) and Relational Attitudes Scale (RAS). | Significantly more Aboriginal girls and women (25.9%) than Caucasian girls and women (8.1%) scored above the BULIT-R cut-off score (>85). An association between marginalization and the severity of bulimic symptoms was found for Aboriginal girls and women. |
| Tessier (2001)*  (66) | Canada | To investigate the prevalence of disordered eating behaviours and attitudes in adolescents in a Northern Canadian community. | Sociodemographic characteristics, ethnic diet and EDI-2. | Canadian Asian adolescents scored above cut-off scores on EDI-2 Body Dissatisfaction, Bulimia and Drive For Thinness subscale more than all other ethnic groups. |
| Boisvert & Harrell (2009)  (68) | Canada | To investigate ethnic and age differences in BMI, body shame and eating pathology. | BMI, EDI (Bulimia, Drive for Thinness and Body Dissatisfaction subscale items) and Objectified Body Consciousness Scale (OBC). | Hispanic women reported more bulimic behaviours than women from all other ethnic groups. No ethnic differences in drive for thinness. |
| Boisvert & Harrell (2012)  (69) | Canada | To investigate ethnicity, spirituality, religiosity, body shame, BMI and age in relation to eating pathology in men. | EDI (Bulimia, Drive For Thinness and Body Dissatisfaction subscale items), Objectified Body Consciousness Scale (OBC), Spiritual Wellbeing Scale (Existential Wellbeing subscale items) and religiosity. | No direct effects of ethnicity on disordered eating. Lower BMI and higher body shame mediated an indirect relationship between Asian ethnicity and disordered eating. Higher BMI and lower spirituality mediated an indirect relationship between Aboriginal status and disordered eating. |
| Buttu (2012)*  (70) | Canada | To explore the role of acculturation, ethnic identity and bicultural stress in disordered eating and body image concerns in Arab Canadians. | Sociodemographic characteristics, BMI, BULIT-R, EAT-26, Body Shape Questionnaire (BSQ), Sociocultural Attitudes Towards Appearance Questionnaire (Internalisation-General subscale), Acculturation Rating Scale of Arab Americans (ARSAA), Multigroup Ethnic Identity Measure (MEIM), Cultural Values Conflict Scale (CVCS) and Cultural Adjustment Difficulties Checklist (Acculturative Distress subscale). | 24% of participants scored above the EAT-26 cut-off score (>20) and 11.2% of participants scored above the BULIT-R cut-off score (>85). Disordered eating associated with Internalization of thinness and body shape concerns. Bicultural stress and conflict but not acculturation or ethnic identity were associated with disordered eating. |
| Boisvert & Harrell (2013)  (71) | Canada | To investigate ethnicity, spirituality, religiosity, body shame, BMI and age in relation to eating pathology in women. | Sociodemographic characteristics, BMI, EDI (Bulimia, Drive for Thinness and Body Dissatisfaction subscale items), Objectified Body Consciousness Scale (OBC), Spiritual Wellbeing Scale (Existential Wellbeing subscale items) and religiosity. | No direct effect of ethnicity on disordered eating. |
| Boisvert & Harrell (2014)  (72) | Canada | To investigate the relationships between ethnicity, SES and urban-rural differences and eating pathology. | BMI, SES and EDI (bulimia, drive for thinness and body dissatisfaction items). | No direct effect of ethnicity on disordered eating. Interaction of ethnicity and income, whereby non-White individuals with higher income had less disordered eating than non-White individuals with lower income. |
| Chan & Owens (2006)  (75) | New Zealand | To examine the role of ethnic identity in the association between perfectionism and disordered eating in Chinese immigrants. | EDI, Positive and Negative Perfectionism Scale (PANPS), Marlow-Crowne Social Desirability Scale (MCSDS) and Multigroup Ethnicity Identity Measure (MEIM). | Greater negative perfectionism associated with higher levels of disordered eating. High positive perfectionism and a strong positive evaluation of other ethnic groups associated with lower scores on EDI Drive For Thinness and Body Dissatisfaction subscales. |
| Oakley-Browne et al. (2006)  (76) | New Zealand | To estimate the lifetime prevalence and projected lifetime risk at 75-years of DSM-IV disorders in New Zealand. | Sociodemographic characteristics, Composite International Diagnosis Interview (CIDI). | Adjusted hazard ratio (controlling for age, sex, education and household income) for estimated lifetime risk of any ED disorder (AN or BN) was 1.6% (95% CI= 1.1-2.4) for Māori and 2.5% (95% CI= 1.6-3.9) for Pacific people. |
| Baxter et al. (2006)  (77) | New Zealand | To describe the prevalence of DSM-IV disorders among Māori in New Zealand. | Sociodemographic characteristics, Composite International Diagnosis Interview (CIDI). | Lifetime prevalence rates of EDs for Māori were 0.7% (95% CI= 0.2-1.6 ) for AN, 2.4% (95% CI= 1.8-3.2) for BN and 3.1% (95% CI= 2.3-4.1) for any ED. |
| Foliaki et al. (2006)  (78) | New Zealand | To describe the prevalence of DSM-IV disorders and treatment contact among Pacific people in New Zealand. | Sociodemographic characteristics, treatment contact, Composite International Diagnosis Interview (CIDI). | Lifetime prevalence rates of EDs for Pacific people were 3.9% (95% CI= 2.7-5.5) for BN and 4.4% (95% CI= 3.1-6.2) for any ED. Rate of 12-month mental health service use was lowest for EDs when compared to other mental health disorders. |
| Ngamanu (2006)*  (79) | New Zealand | To compare levels of body dissatisfaction and eating pathology in Māori and Pakeha women and examine their association with ethnic identity. | Sociodemographic characteristics,  EAT-26, Multigroup Ethnicity Identity Measure (MEIM) and Figure Rating Scale (FRS). | Eating pathology and body image dissatisfaction did not differ between ethnic groups. N= 2 Māori, N= 1 Māori and Pakeha and N=3 Pakeha scored above EAT-26 cut-off (>20). Ethnic identity was not associated with eating pathology or body image satisfaction. |
| Jenkins (2007)*  (80) | New Zealand | To determine the prevalence of ED symptomology and body dissatisfaction in Chinese women and examine their association with length of time living in New Zealand. To assess the appropriateness of the EAT as a diagnostic tool. | Sociodemographic characteristics, BMI, ET-26, EAT-40, SEED, Eating Disorder Beliefs Questionnaire (Acceptance by Others and Self-Acceptance subscales), Perceived Sociocultural Pressure Scale and body image. | Compared to students from other ethnic groups, Chinese students had a greater fear of weight gain, disordered eating and body dissatisfaction. 15.9% of Chinese students and 11.3% of 'other' students scored above EAT-40 cut-off score (>30). 15.9% of Chinese students and 16.9% of 'other' students scored above the EAT-26 cut-off score (>20). Length of time in New Zealand had no effect on disordered eating and body dissatisfaction in Chinese students. EAT-26 and EAT-40 found not to be appropriate for use in Chinese samples. |
| Chan et al. (2010)  (81) | New Zealand | To examine the role of ethnic identity in the association between perfectionism and disordered eating in Korean Immigrants. | Sociodemographic characteristics, EDI, Positive and Negative Perfectionism Scale (PANPS), Marlow-Crowne Social Desirability Scale (MCSDS) and Multigroup Ethnicity Identity Measure (MEIM). | Positive and negative perfectionism were associated with ED symptoms. Strong Korean ethnicity identity moderated the association between negative perfectionism and EDI Body Dissatisfaction subscale scores in boys and men, but not girls and women. |
| Dolan et al. (1990)  (93) | UK | To compare eating attitudes and behaviours and perception of body weight and shape in women across three ethnic groups. | Sociodemographic characteristics, EAT-26, Body Shape Questionnaire (BSQ) and Hospital Anxiety and Depression Scale (HADS). | Highest levels of disordered eating in Asian women. 7.0% of Afro-Caribbean sample, 16.0% of Asian sample and 8.5% of Caucasian sample scored above cut-off on EAT-26 (>20). No ethnic differences in body weight and shape concerns. Association between disordered eating and anxiety and depression found only for Caucasian women. |
| Wardle & Marsland (1990)  (94) | UK | To investigate weight concern and dieting in schoolchildren from varying cultural and socioeconomic backgrounds. | Sociodemographic characteristics, DEBQ and body image concerns. | Dieting and restrained eating was found across all social and ethnic groups but was most common in White girls from higher social status schools. Controlling for SES and BMI, White and Asian children scored higher on the DEBQ Restrained Eating subscale then Black children. |
| Mumford et al. (1991)  (96) | UK | To assess the validity of the EAT-26 and BSQ in a sample of South Asian schoolgirls. | BMI, EAT-26, EDE, Body Shape Questionnaire (BSQ) and cultural orientation. | Factor analysis of the EAT-26 and BSQ indicated cross-cultural comprehension and validity. Higher levels of disordered eating in South Asian schoolgirls compared to Caucasian schoolgirls. 12.3% of Asian sample and 8.7% of Caucasian sample scored above cut off on EAT-26 (>20). Higher EAT-26 and BSQ scores and BN diagnosis in the South Asian sample were associated with a "traditional" orientation. No significant differences in EAT-26 score for South Asian girls according to place of birth or religion. |
| Wardle et al. (1993)  (97) | UK | To investigate weight concerns and dieting in Asian and White girls and women. | Sociodemographic characteristics, DEBQ, size judgements, body parts perception, Figure Rating Scale (FRS), Body Shape Questionnaire (BSQ), reading and viewing habits. | Compared to White participants, Asian participants reported less body dissatisfaction and scored lower on the DEBQ Restrained Eating subscale. However, ethnic differences were reduced and, in some cases, eliminated when accounting for lower BMI of Asian participants. |
| Ahmad et al. (1994a)  (99) | UK | To investigate the role of perceived parental control in the relationship between ethnicity and eating pathology in young girls. | Sociodemographic characteristics, EAT-26, Body Satisfaction Scale (BSS), Parental Bonding Instrument (PBI). | Higher levels of disordered eating in Asian schoolgirls compared to Caucasian schoolgirls. However, only differences in EAT-26 Bulimia subscale scores were significant. Greater levels of perceived maternal control among Asian schoolgirls accounted for some of the difference in bulimic scores. |
| Ahmad et al. (1994b)  (100) | UK | To investigate the role of religion and gender in the relationship between ethnicity and eating pathology in children. | Sociodemographic characteristics, EAT-26, Body Satisfaction Scale (BSS). | Compared to Caucasian and Hindu adolescents, Muslim adolescents, had significantly higher bulimic eating attitudes but greater body satisfaction. Eating attitudes and behaviours were particularly poor for Muslim boys who had higher EAT-26 total, and Oral Control subscale scores compared to Hindu and Caucasian boys. |
| Furnham & Patel (1994)  (101) | UK | To investigate the influence of perceived integration into British culture and resentment towards parents on the relationship between ethnicity and eating pathology. | Sociodemographic characteristics, EAT-26, BEQ and perceived cultural integration. | No significant difference in disordered eating or vomiting between Asian and Caucasian girls. Among Asian girls weak associations were found between higher levels of resentment towards family and less integration into British culture and disordered eating behaviours. |
| Hill & Bhatti (1995)  (104) | UK | To examine the relationship between perception of body weight and shape and dietary restraint in Asian girls. | BMI, DEBQ, body esteem, Body Cathexis Scale, body figure preferences and cultural orientation. | Asian girls reported significantly higher levels of dietary restraint compared to Caucasian girls. Girls with the highest levels of dietary restraint had lower body satisfaction and self-esteem. An additional association between high levels of dietary restraint and a "traditional" family environment was found for Asian girls. |
| McCourt & Waller (1995)  (105) | UK | To investigate the effects of perceived parental control on eating pathology at different ages across adolescence. | Sociodemographic characteristics, EAT-26 and Parental Bonding Instrument (PBI). | Compared to Caucasian adolescents, Asian adolescents had higher levels of disordered eating at all ages. Greater disordered eating in Asian adolescents was partially explained by higher levels of perceived maternal control, this association was strongest for older girls aged 15-16 years. |
| Waller et al. (1995)  (106) | UK | To examine the nature and patterns of bulimic eating behaviours in adolescent schoolgirls. | Sociodemographic characteristics and BITE. | No significant ethnic differences in overall or subscale BITE scores. However, significant differences in the presence and severity of specific bulimic behaviours and attitudes were found. Asian girls were more likely to report food dominating their lives and eating differently in public and private and had a greater frequency of fasting (even when accounting for religious reasons). Whilst Caucasian girls had a greater frequency of bingeing and were more likely to report bingeing when alone. |
| Button et al. (1998)  (109) | UK | To investigate possible differences in eating pathology, associated psychological difficulties and illness perception and help-seeking in young women from different ethnic backgrounds. | Sociodemographic characteristics, BMI, EAT-26, Rosenberg Self Esteem Scale (RSES), Hospital Anxiety and Depression Scale (HADS). Vignettes to assess illness perception and help-seeking behaviours for severe recurrent headaches, depression and disordered eating. | No significant differences in eating attitudes and behaviours between ethnic groups, aside from self-induced vomiting for weight loss being more common in Caucasian women (11.5% vs. 1.4% Asian & 0% Black). No differences in illness perception or help-seeking behaviours according to ethnicity. Two-thirds of Asian women reported religion affected their diet. |
| Ogden & Elder (1998)  (110) | UK | To examine the influence of ethnicity and family status on eating behaviours and body dissatisfaction. | Sociodemographic characteristics, DEBQ, Body Shape Questionnaire (BSQ) and acculturation. | White women reported greater levels of restrained eating. Ethnicity x family status interaction for calorie concern whereby, White daughters had the greatest concern over calorie content in food. No relationship between acculturation and eating behaviour or between mother and daughter eating behaviours. |
| Ogden & Chanana (1998)  (111) | UK | To investigate the role of family in the relationship between ethnicity and weight concern in young women. | Sociodemographic characteristics, DEBQ, Body Shape Questionnaire (BSQ) and family values and beliefs. | No differences in restrained eating or body dissatisfaction between Asian and White daughters. Ethnicity was not a significant predictor of restrained eating across women. Cultural values and beliefs rather than ethnicity per se which influences eating behaviours. |
| Furnham & Husain (1999)  (112) | UK | To investigate the role of parental conflict and overprotection in its relationship between ethnicity and eating pathology in young women. | Sociodemographic characteristics, BMI, EAT-26, Parental Bonding Instrument (PBI) and parental conflict. | No significant differences in disordered eating behaviours between Asian and White women. EAT-26 scores in Asian women correlated with parental conflict over 'Going Out' and 'Choice of Friends', but no correlations were found between parental overprotection and EAT-26 scores. 7.3% of Asian women and 13.4% of White women scored above cut-off point on EAT-26 (>20). |
| Furnham & Adam-Saib (2001)  (114) | UK | To examine intra-Asian differences in eating attitudes and body dissatisfaction and assess their relationship with parental control in second-generation Bengali, Indian, Pakistani adolescents. | Sociodemographic characteristics, BMI, EAT-26, Body Satisfaction Scale (BSS) and Parental Bonding Instrument (PBI). | Compared to White counterparts, the (total) Asian sample had significantly higher scores for disordered eating (EAT-26 total and Oral Control subscale score) and parental overprotection but not body dissatisfaction. Bengali girls had significantly higher EAT-26 total, and Dieting subscale scores compared to all other ethnic groups and higher Oral Control subscale scores compared to Indian and White girls (but not Pakistani girls). For all girls body dissatisfaction but not parental overprotection predicted disordered eating. |
| Mujtaba & Furnham (2001)  (115) | UK | To explore the relationship between disordered eating and parental conflict and overprotection in late adolescent girls across three cultures. | Sociodemographic characteristics, BMI, EAT-26, Body Shape Belief Scale (BSBS), Parental Bonding Instrument (PBI). Interviews to elaborate on questionnaire responses. | British Asian women had highest scores for disordered eating, parental conflict and parental overprotection scores. For the total sample, disordered eating behaviours and attitudes were associated with parental conflict and overprotection. |
| Thomas et al. (2002)  (116) | UK | To examine the influence of ethnicity, gender, socioeconomic status, self-esteem and emotion on eating attitudes and behaviours in adolescents. | Sociodemographic characteristics, BMI, EAT-26, Rosenberg Self Esteem Scale (RSES) and Angold Mood and Feelings Questionnaire. | Compared to White counterparts, Asian and mixed-race students but not African Caribbean students had significantly greater disordered eating. Ethnicity was a significant and independent predictor of disordered eating. Adjusted odds ratio for having an EAT-26 score above the cut-off (>20) was 2.44 (95% CI 1.0-6.0) for Asian and Muslim students and 4.86 (95% CI 1.3-18.6) for mixed-race students, compared to White counterparts. |
| Dogra et al. (2013)  (125) | UK | To establish the prevalence rates of mental health problems in Indian and White adolescents living in England. | Sociodemographic characteristics, SCOFF, Strengths and Difficulties Questionnaire (SDQ) and Short Mood and Feelings Questionnaire (SMFQ). | Indian adolescents had lower rates of mental health problems compared to White counterparts with the exception of disordered eating, where no ethnic differences were found. 8% of both Indian and White adolescents scored above SCOFF cut-off (≥3). |
| Solmi et al. (2014)  (126) | UK | To estimate the prevalence of disordered eating, its sociodemographic and psychological correlates and associated patterns of service use in a community sample. | Sociodemographic characteristics, SCOFF, (CIS-R), (SAPASP, (PC-PTSD), AUDIT, drug use, smoking and suicidal ideation and/or attempt. | Disordered eating was more common in minority ethnic individuals. 16.1% of 'mixed or other', 14.8% of Asian, 12.8% of Black and 7.9% of White participants scored above SCOFF cut-off (≥ 2). In adjusted analyses only 'other' ethnic background was a significant predictor of disordered eating (OR= 1.8, 95% CI 1.1-3.0). In adjusted analyses when compared to White participants, Asian ethnicity was associated with purging, loss of control eating and pre-occupation with food and 'other' ethnicity was associated with purging, body image distortion and preoccupation with food. There was no association of Black ethnicity with any of the SCOFF questions. |
| Swami (2016)  (128) | UK | To examine the change in risk for developing EDs in Malay students studying in the UK | EDI-3 (Drive for Thinness, Body Dissatisfaction and Bulimia subscales) completed 2 months before arriving in the UK (Time 1) and 4 months after arriving in the UK (Time 2). Measures of sociocultural adjustment, cultural distance between home and host cultures and perceived discrimination also completed at Time 2. | EDI-3 Drive For Thinness, Body Dissatisfaction and Bulimia subscales were significantly higher at Time 2. Poor sociocultural adjustment and greater perceived discrimination predicted greater risk of disordered eating behaviours. |

Abbreviations: BEQ – Binge Eating Questionnaire; BITE – Bulimic Investigatory Test, Edinburgh; BMI – Body Mass Index; DEBQ – Dutch Eating Behaviour Questionnaire; CI – Confidence Interval; EAT – Eating Attitudes Test; EDE-S – Eating Disorder Examination Screening Version; EDI – Eating Disorder Inventory; EDI-SC – Eating Disorder Inventory Symptom Checklist; OR – Odds Ratio; SES – Socioeconomic Status

* = Thesis

## **Table S7** Two-stage prevalence studies

| **First Author & Year of Publication** | **Country** | **Aim/Focus** | **Data Collected** | **Summary of findings** |
| --- | --- | --- | --- | --- |
| Nasser (1986)  (86) | UK | To estimate the prevalence of disordered eating attitudes in Arab women. | EAT-40 and diagnostic interview using ‘The Eating Interview’ | 22% of London sample vs. 12% of Cairo sample scored above cut-off on EAT-40 (>30). In the London sample, N= 6 women met diagnostic criteria for BN and N= 5 for partial AN syndrome. No women from Cairo sample met diagnostic criteria. |
| Mumford & Whitehouse (1988)  (87) | UK | To estimate the 1-year prevalence of AN and BN in South Asian and Caucasian schoolgirls. | EAT-26 and diagnostic interview using EDE | One-year prevalence of BN was significantly higher in South Asian schoolgirls (3.4%) compared to Caucasian schoolgirls (0.6%). AN diagnosed in N= 1 Asian girl but none of the Caucasian girls. |
| Reiss (1996)  (107) | UK | To compare bulimic attitudes and behaviours and associated psychological difficulties in African Caribbean and White British women. | BITE, General Health Questionnaire (GHQ-28) and diagnostic interviews | African-Caribbean women had significantly greater disordered eating attitudes and behaviours compared to White women. African-Caribbean women were more likely to report feelings of guilt and failure when overeating and being dominated by food. N= 6 diagnosed with BN (N= 1 African, N= 1 African-Caribbean, N= 1 Asian, N= 1 'other/Mixed race', N= 2 White). |
| Mumford & Choudry (2000)  (113) | UK | To examine the association between body dissatisfaction and eating attitudes across ethnic groups. | EAT-26, BSQ and diagnostic interviews | Compared to 49% of women in Pakistan, only 10% of British South Asian women and White British women scored above EAT-26 cut-off (>20). N= 3 women in Pakistan diagnosed with BN. N= 1 British South Asian woman diagnosed with partial BN. Interview rates too low to calculate prevalence in London sample. Negative correlation between body dissatisfaction and disordered eating behaviours and attitudes in all ethnic groups. |
| Bhugra & Bhui (2003)  (117) | UK | To explore the prevalence of bulimic behaviours and their association with acculturation across three different ethnic groups. | BITE, acculturation, sociodemographic characteristics, BMI, eating patterns and identity and diagnostic interviews | Asian students had highest overall BITE score compared to all other ethnic groups, though difference in overall BITE scores across ethnic groups were insignificant. Asian students were more likely to think about food and engage in fasting or compulsive eating. African-Caribbean students were least likely to view themselves as 'normal eaters' and most likely to eat sensibly in front of others but make up for it in private. Acculturation was not associated with bulimic attitudes and behaviours for any ethnic group. No individuals diagnosed with DSM-III-R ED. |
| Solmi et al. (2016)  (10) | UK | To estimate the prevalence of DSM-5 EDs and investigate associated co-morbidity and service use in a community sample. | SCOFF and diagnostic interviews using SCID-I-NP ED section | Point prevalence of any ED was 4.4% (3.6% BED, 0.8% BN) and 7.4% when including sub-threshold diagnoses (0.6% PD, 2.4% OSFED). No cases of AN identified. No significant differences in prevalence across ethnic groups. White participants had highest prevalence of BED. 1/3 of all BN cases occurred in Black participants. |

Abbreviations: AN – Anorexia Nervosa; BED – Binge Eating Disorder; BITE – Bulimic Investigatory Test, Edinburgh; BN – Bulimia Nervosa; DSM – Diagnostic and Statistical Manual; EAT – Eating Attitudes Test; ED – Eating Disorder; EDE – Eating Disorder Examination; OSFED – Other Specified Feeding or Eating Disorder; SCID-I-NP - Structured Clinical Interview for DSM-IV Axis I Disorders Non-patient Edition.

## **Table S8** Other quantitative studies

| **First Author & Year of Publication** | **Country** | **Aim/Focus** | **Key Data Collected** | **Summary of findings** |
| --- | --- | --- | --- | --- |
| Davey (2012)*  (82) | New Zealand | To evaluate the effectiveness of two pre-treatment motivation groups (Motivation Group vs. Motivation and Education Group). | Efficacy of group assessed using: EDE-Q, Beck Depression Inventory (BDI), Details and Flexibility (DFLEX), Motivational Stages of Change (MSOC) and Change Continuum (CC). | Differences in treatment attendance according to ethnicity. Māori patients had lowest attendance. 16.7% of Māori patients, 33.5% of New Zealand European patients and 50% of Asian patients completed all four group sessions. Difference in outcome measures pre- and post-treatment not reported by ethnicity. |
| Currin et al. (2007)  (119) | UK | To examine the influence of clinical (i.e., weight status, diabetes history) and non-clinical features (i.e., gender, ethnicity) of case presentation on the diagnosis and treatment of EDs in primary care. | Primary diagnosis and treatment course selected for a case vignette depicting an ED presentation. | No significant differences in primary diagnosis or choice of treatment given to case vignettes based on ethnicity. There was a non-significant trend whereby compared to White case vignettes, Afro-Caribbean case vignettes were more likely to be offered a follow-up appointment then a direct referral to secondary/specialist mental health services. |
| Chaudary (2017)*  (129) | UK | To examine the influence of ethnicity on clinical recognition of disordered eating patterns and treatment recommendations in IAPT services. | Primary diagnosis, treatment course and barriers to treatment engagement selected for each case vignette (i.e., restrictive eating, binge eating and anxiety/depression vignette). | White case vignette more likely to be given a diagnosis of AN and South Asian case vignette more likely to be given a diagnosis of BN. No influence of ethnicity on treatment recommendations. Non-significant trend where the South Asian case vignette was offered less intensive treatment (Step 2 – guided self-help) compared to the White case vignette (Step 3 - CBT) for the restrictive eating presentation. Family context and treatment drop-out were greater concerns for White case vignette. |
| Petkova et al. (2019)  (132) | UK | To estimate the incidence of DSM-5 AN in young people attending second care services in the UK and Ireland. | Cases identified through the Child and Adolescent Psychiatry Surveillance System (CAPPS). | Incidence of AN was 14 per 100,000. Majority of incident cases were White (92%) and girls (91%). |
| Dalton et al. (2024)  (136) | UK | To assess the feasibility, acceptability and effectiveness of virtually delivered guided self-help intervention (GSH) for adults with bulimic EDs. | Treatment uptake, attendance and completion (feasibility and acceptability). Weight, frequency of ED behaviours, ED-15 (effectiveness). | Compared to White patients, minority ethnic patients were more likely to decline or drop out of treatment. Prioritisation of weight loss, conflicting commitments and previous experiences of poor care highlighted as reasons for discontinuing treatment. Effectiveness of treatment not reported by ethnicity. |

Abbreviations: AN – Anorexia Nervosa; DSM – Diagnostic and Statistical Manual; EAT – Eating Attitudes Test; ED – Eating Disorder; EDE-Q – Eating Disorder Examination Questionnaire; IAPT – Improving Access to Psychological Therapies

* = Thesis
